# Why Are Autism Spectrum Conditions More Prevalent in Males?

**DOI:** 10.1371/journal.pbio.1001081

**Published:** 2011-06-14

**Authors:** Simon Baron-Cohen, Michael V. Lombardo, Bonnie Auyeung, Emma Ashwin, Bhismadev Chakrabarti, Rebecca Knickmeyer

**Affiliations:** 1 Autism Research Centre, Department of Psychiatry, University of Cambridge, Cambridge, United Kingdom; 2 Centre for Integrative Neuroscience and Neurodynamics, School of Psychology and Clinical Language Sciences, University of Reading, Reading, United Kingdom; 3 Department of Psychiatry, University of North Carolina–Chapel Hill, Chapel Hill, North Carolina, United States of America

## Abstract

Autism Spectrum Conditions (ASC) are much more common in males, a bias that may offer clues to the etiology of this condition. Although the cause of this bias remains a mystery, we argue that it occurs because ASC is an extreme manifestation of the male brain. The extreme male brain (EMB) theory, first proposed in 1997, is an extension of the Empathizing-Systemizing (E-S) theory of typical sex differences that proposes that females on average have a stronger drive to empathize while males on average have a stronger drive to systemize. In this first major update since 2005, we describe some of the evidence relating to the EMB theory of ASC and consider how typical sex differences in brain structure may be relevant to ASC. One possible biological mechanism to account for the male bias is the effect of fetal testosterone (fT). We also consider alternative biological theories, the X and Y chromosome theories, and the reduced autosomal penetrance theory. None of these theories has yet been fully confirmed or refuted, though the weight of evidence in favor of the fT theory is growing from converging sources (longitudinal amniocentesis studies from pregnancy to age 10 years old, current hormone studies, and genetic association studies of SNPs in the sex steroid pathways). Ultimately, as these theories are not mutually exclusive and ASC is multi-factorial, they may help explain the male prevalence of ASC.

## Is There Really a Male Bias?

The diagnosis of classic autism and Asperger Syndrome (AS), known as Autism Spectrum Conditions (ASC), rests on difficulties in reciprocal social interaction and communication, alongside strongly repetitive behavior and unusually narrow interests [1]. The prevalence of ASC is estimated to be 1% [2],[3]. A diagnosis of classic autism, unlike AS, also requires the presence of additional learning difficulties and language delay. ASC is neurobiological, evidenced by atypical brain development in structure and function [4]. ASC is also genetic [5],[6] though not without some interaction with environmental influences.

ASC is strongly biased towards males [7], with ratios of 4∶1 (male∶female) for classic autism [8] and as high as 11∶1 in individuals with AS [9]. The specific factors responsible for the higher male prevalence in ASC remain unclear. ASC is not the only neurodevelopmental condition more common among males—a greater prevalence in males versus females is also seen in Attention Deficit and Hyperactivity Disorder (ADHD), dyslexia, conduct disorder (CD), specific language impairment, Tourette Syndrome, and Learning Difficulties (see Table 1) [10].

**Table 1:** Male biased sex ratios in other neurodevelopmental conditions.

**1**	**Attention Deficit Hyperactivity Disorder (ADHD).** The ratio of males to females with ADHD is high in clinic samples (up to 10∶1) [106],[107]. However, it drops to 2∶1 to 4∶1 in community samples [108],[109],[110] and the majority of studies in adults show no significant effect of sex on prevalence [111]. This suggests that the biased sex ratios observed in ADHD may result from referral bias rather than a biological mechanism.
**2**	**Conduct Disorder (CD).** Males are two to four times more likely to develop CD than females [112], though no sex difference was observed in the recent NHANES study [110]. This discrepancy probably reflects the observation that while sex differences are not pronounced in adolescent-limited antisocial behavior, the male∶female ratio for early-onset, life-course-persistent antisocial behavior is 10∶1 or greater [113].
**3**	**Dyslexia/Reading Disability (RD).** Early research suggested that there was a significant excess of males with RD, but this view has been challenged as reflecting referral bias and subjective methods of assessment [114]. It is clear that ascertainment bias does inflate the true prevalence of RD in males, but a review of existing studies suggests that there is a slightly skewed gender ratio, between 1.7 and 2.00 [115].
**4**	**Specific Language Impairment (SLI).** While many early studies reported a male biased sex ratio of between 2∶1 and 3∶1 [116] for SLI, it has been suggested that this reflects ascertainment bias [114]. Epidemiological studies have identified equivalent numbers of males and females meeting diagnostic criteria [117] or increased prevalence in females [118].
**5**	**Tourette Syndrome (TS).** TS shows a male to female ratio of between 4∶1 and 6∶1 [119]. It is notable that 50%–90% have comorbid ADHD, particularly in clinic populations, which may contribute to the biased ratio.

However, the male bias is much more pronounced in ASC, especially in the case of AS. This male bias could simply reflect the difficulty of diagnosing AS in females. Though classic autism would not be missed in females, AS could be if it presented as some other condition, such as anorexia [11] or borderline personality disorder [12], both of which involve the exercise of excessive control over the environment or other people, and a certain degree of a self-centeredness. Equally, AS in females could be under-diagnosed if females are more motivated to learn to conform socially or have better imitation skills that allow them to “pretend to be normal” [13]. Finally, this male bias might reflect the inability of the widely used diagnostic instruments (the Autism Diagnostic Observation Schedule (ADOS) or Autism Diagnostic Interview-Revised (ADI-R)) to detect the more subtle ways in which AS may present in females.

While these explanations of mis- or under-diagnosis may explain part of the male bias, there may also be *biological* reasons for the male bias in ASC. We argue that the bias can be understood as an extreme expression of the psychological and physiological attributes of the male brain; that is, males need only slight psychological and physiological changes to exhibit ASC while females would require more, thus making ASC rarer in females. What factors might favor overdevelopment of male characteristics? One possible biological mechanism could be the masculinizing effect of fetal testosterone (fT). Two other possibilities include the X- and Y-linked theories and the reduced autosomal penetrance theory (which posits that females harbor fewer ASC-related mutations on autosomal chromosomes). Future research will help to resolve the validity or flaws of these theories, which for now remain neither fully confirmed nor refuted. Here, we lay out some of the evidence for these theories in explaining the male bias in ASC.

## Is ACS an Extreme Expression of the Male Brain?

The Extreme Male Brain (EMB) theory of autism extends the Empathizing-Systemizing (E-S) theory of typical sex differences [14], which proposes that females on average have a stronger drive to *empathize* (to identify another person's thoughts and feelings and to respond to these with an appropriate emotion), while males on average have a stronger drive to *systemize* (to analyze or construct rule-based systems). Whilst sociologists still debate if there are any sex differences at all, and if so whether these are purely the result of cultural conditioning, biologists have long known from animal research that sex differences in behavior exist in primates and are influenced by biology as well as the environment.

On the Empathy Quotient (EQ) [15] typical females score higher than typical males who score higher than those with ASC [15]. On the Systemizing Quotient (SQ), individuals with ASC score higher than typical males who score higher than typical females [16]–[18]. Additional psychological evidence (summarized in Table 2 and in Text S1) shows that—irrespective of the direction of sex difference—people with autism show an extreme of the male profile. Note that the EMB theory does not state that all psychological sex differences will be exaggerated in ASC—only those relating to empathy and systemizing.

**Table 2:** A summary of the psychological evidence for the Extreme Male Brain (EMB) theory (see Text S1 for a fuller discussion).

Psychological Measure	Autism>Male>Female	Female>Male>Autism	Key References
Adolescent AQ	✓		[120]
Adult Autism Spectrum Quotient (AQ)	✓		[104],[121]–[124]
Adult Systemizing Quotient (SQ)	✓		[16]
Child AQ	✓		[125]
Child SQ	✓		[126]
Childhood Autism Spectrum Test (CAST)	✓		[127]–[130]
Embedded Figures Test	✓		[131],[132]
Intuitive Physics Test	✓		[133],[134]
Social Responsiveness Scale	✓		[135],[136]
Quantitative Checklist for Autism in Toddlers (Q-CHAT)	✓		[137]
Adult Empathy Quotient (EQ)		✓	[15]
Child EQ		✓	[126]
Faux Pas Test		✓	[138]
Friendship and Relationship Questionnaire (FQ)		✓	[139]
Reading the Mind in the Eyes		✓	[140]
Social Stories Questionnaire (SSQ)		✓	[133]

### Sexual Dimorphism in the Human Brain

Additional support for the EMB theory of ASC comes from evidence of neural sexual dimorphism across development. Some key examples of typical sexual dimorphism reveal an extreme of the typical male profile in the neurodevelopment of ASC [19]. However, one caveat to keep in mind is that just as all *psychological* sex differences do not constitute an exaggerated form of maleness in ASC, neither do all *neural* differences. Indeed, given that the EMB theory is defined at the psychological level, we should expect only a narrow set of neural sex differences will be involved in such hyper-masculinization in ASC. A key finding supporting this prediction is that infant males on average have a larger brain than females [20] and children with autism have even larger brains early in life right around the time they would typically receive a diagnosis (2–4 years) [21]. In addition, independent of global differences in brain size, the amygdala in typical males tends to be larger than in females [22], and early in development the amygdala in autism is even more enlarged than that observed in typical males [23]–[25]. In addition to such *structural* sexual dimorphism in the brain, exaggeration of neural sexual dimorphism extends to brain *function* and corroborates predictions from the EMB theory (see Table 3 and Text S1 for fuller discussion) [26]–[29].

**Table 3:** A summary of the evidence consistent with the EMB theory at the neural level (see Text S1 for a fuller discussion).

Brain Region	Autism>Male>Female	Female>Male>Autism	Key References
**Structure**			
Total brain volume	✓		[20],[141]–[143]
Amgydala	✓		[22]–[25],[144]–[150].
Corpus callosum		✓	[151],[152]
Perisylvian language areas (Heschl's gyrus/planum temporale)		✓	[22],[153]–[156]
L>R asymmetry in planum temporale		✓	[22],[154],[157]–[160]
Lateral fronto-parietal cortex		✓	[144],[145],[147],[150],[156],[161]–[165]
**Function**			
Default Mode Network Connectivity		✓	[166],[167]
Embedded Figures fMRI		✓	[27]–[29],[168]
Reading the Mind in the Eyes task fMRI		✓	[26],[28]

The set of striking findings of hyper-masculinization in ASC at three simultaneous levels (cognitive, neuroanatomy, and neural function) raises the question as to which biological mechanism(s) are involved. Two plausible mechanisms that could give rise to sexual dimorphism, hyper-masculinization, and/or the absence of typical sexual dimorphism at the levels of brain, cognition, and behavior are the “organizing” effects of fetal testosterone (fT) [30]–[32] and X- or Y-linked genetic factors. We review these three interesting hypotheses, since these may also have relevance to the sex ratio in ASC. These are not proposed as complete explanations for ASC, since ASC is recognized to be multi-factorial, but they may form an important part of the explanation.

## What Might Cause an Extreme Male Brain?

### The Fetal Testosterone (fT) Theory

#### Fetal androgens affect the brain: Evidence from animal and human studies

Animal studies, especially in rodents, confirm that early exposure to androgens (such as testosterone) acts on the brain to produce sex differences in behavior, cognition, brain structure, and function (see Text S1 for more discussion of work with animals) [31]–[33]. It is widely accepted that fT exposure also affects brain development and behavior in humans. Human males experience a surge in fT between weeks 8 to 24 of gestation [34]–[36], reaching almost pubertal levels. There is also a second surge soon after birth (here called “neonatal testosterone,” or nT). Usually the levels remain high and then drop to barely detectable levels by 4–6 months [37], until the third surge at puberty. Whilst the third surge is understood to be controlling the onset of puberty, the function of first surge (fT) is believed to play a major role in brain masculinization.

While direct manipulation of hormones as has been conducted in animal studies is unquestionably unethical in human fetuses and infants, alternative research strategies include relating individual variation in amniotic fT exposure to later development [38], or studying people in whom—for medical reasons—the sex hormones are higher or lower than expected for a person's sex [39], and using proxy measures of fT exposure. Here we review evidence from studies of cognitive traits relevant to ASC and their relationship with amniotic fT. (Evidence from disorders of sexual differentiation and from proxy measures of fT exposure is presented in the Text S1.)

#### Fetal androgens affect ASC traits: evidence from amniotic fluid testosterone

fT can be measured in amniotic fluid, obtained during routine amniocentesis. Because amniocentesis is typically performed during the second trimester of pregnancy (usually 14–20 weeks of gestation), when serum testosterone peaks in male fetuses, it offers a unique opportunity to compare fT with ASC traits. There is a well-documented large sex difference in amniotic androgen levels [40]–[44]. The origin of androgens in amniotic fluid appears to be the fetus itself, and testosterone obtained in amniotic fluid is thought to be a good reflection of the levels in the fetus [38]. In the Cambridge Fetal Testosterone Project, initiated by our group in 1998, children whose mothers had amniocentesis during pregnancy (but who were otherwise developing normally) have been followed up after birth every year or two and are now approximately 11 years of age [34].

Evidence that amniotic fT affects individual differences in cognitive development in typically developing children (but with clear relevance to ASC) includes the following: fT is *inversely* associated with frequency of eye contact at 12 months old [45] and with size of vocabulary development at 18 and 24 months [46]. fT is also *inversely* associated with quality of social relationships at 48 months [47] and with empathy at 48 and 96 months [48],[49]. In contrast, amniotic fT is *positively* associated with narrow interests at 48 months [47], with “systemizing” at 96 months [18], and with performance on the Embedded Figures Test (EFT) as a measure of attention to detail at 96 months [50]. These are all behaviors that show sexual dimorphism, but critically, these fT effects are often found within one sex as well as when analyzing the sexes combined. The finding of a consistent inverse correlation between fT and social domains, and a consistent positive correlation between fT and non-social domains, across development, is striking and suggests these are real effects which substantiate the notion that fT plays an “organizational” role in development.

In the first study to directly assess if fT affects not just human cognition but also human brain structure, we found that increasing levels of fT are associated with increasing rightward asymmetry in the thickness of one subsection of the corpus callosum, the isthmus [51]. This is interesting since the isthmus projects to posterior parietal and superior temporal cortices, which are integral for language and visuospatial ability and are known to be sexually dimorphic in lateralization, structure, and function (see Text S1).

All of the above behavioral domains (eye contact, language development, quality of social relationships, narrow interests, empathy, systemizing, and embedded figures/attention to detail) and brain structure show sexual dimorphism and appear hyper-masculinized in ASC, raising the possibility that fT may play a role in the development of ASC itself. Three recent experiments have confirmed a positive correlation between fT levels and the number of autistic traits a child shows in toddlerhood [52] and in later childhood [53]. The Cambridge Fetal Testosterone Project has too few children (currently *n* = 635 are enrolled) to test whether fT is elevated in those who later are diagnosed with ASC, but testing for a *direct* association between fT levels and diagnosed ASC will be possible in our ongoing collaboration with the Danish Biobank, which has tens of thousands of amniotic samples, with adequate power to test this hypothesis. Using a different line of evidence, a number of studies have found also *current* androgen dysregulation in ASC or in their relatives, or androgen-related genes being associated with ASC (see Table 4 for a summary of the evidence for the fT/androgen theory).

**Table 4:** Evidence for the effect of sex steroids in autism (see Text S1 for a fuller discussion).

Evidence	Key References
**From typically developing children**	
Eye contact is inversely related to fT	[45]
Quality of social relationships are inversely related to fT	[47]
Vocabulary size is inversely related to fT	[46]
Empathy is inversely related to fT	[48],[49]
Autistic traits are positively associated with fT	[52],[53]
Restricted interests are positively associated with fT	[47]
Systemizing is positively associated with fT	[18]
Rightward asymmetry in the isthmus of the corpus callosum is positively associated with fT	[51]
**From people with ASC**	
10 genes involved in sex steroid synthesis, transport, and/or metabolism associated with AS or AQ or empathy: *HSD11B1*, *LHCGR*, *CYP17A1*, *CYP19A1*, *SCP2*, *CYP11B1*, *ESR1*, *ESR2*, *HSD17B4*, *HSD17B2*	[169]
Timing of puberty: Boys with ASC enter puberty earlier. Girls with ASC enter puberty later	[170]–[172]
Testosterone related medical conditions in women with ASC and their mothers (e.g., PCOS, breast and ovarian cancers, acne)	[172]
Testosterone related characteristics in women with ASC and their mothers	[172],[173]
Lower 2D∶4D ratio in ASC, and parents	[174]–[176]
SRD5A1 and AR genes associated with ASC	[177],[178]
Decreased expression of RORA gene and aromatase in post-mortem frontal and cerebellar tissue	[179],[180]
Females with Congenital Adrenal Hyperplasia (CAH) have elevated AQ	[181]
Testosterone levels are elevated in ASC	[182]
Androstenedione levels are elevated in ASC	[183]

Although some studies have failed to support a role for testosterone in ASC (and most of these have not been able to study fT specifically), the studies reported above suggest that fT is implicated in the biased sex ratio seen in ASC. However, alternative models exist which could also explain the excess of males with ASC. In the final part of this article we review the main contender, the X chromosome theory. For completeness, we also briefly review the Y chromosome theory and the reduced autosomal penetrance theory.

### The X Chromosome Theory

The X chromosome contains more genes expressed in the brain than the other chromosomes [54]. In addition, more than 10% of people with learning difficulties show an X-linked pattern of inheritance [55], involving mutations in over 90 different X-linked genes [56],[57]. Individuals with X-linked learning difficulties may also have ASC, the best-known example being Fragile X Syndrome, where 46% of males and 16% of females carrying the full mutation also have ASC [58].

On the face of it, the biased sex ratio in ASC would therefore be parsimoniously explained by an X chromosome theory. A problem for this theory is that the majority of linkage and association studies of ASC have failed to find regions of interest on the X chromosome [59]–[72]. A related problem for this theory is that in the three recent genome-wide studies of copy number variation (CNV) in individuals with ASC that identified mutations affecting the X chromosome, this was only true in a very small minority of cases. This suggests X-linked mutations are only occasionally seen in ASC and therefore cannot account for the large majority of cases. A final problem for the X-linked theory is that other large CNV scans have reported no significant findings on the X chromosome [67],[73]–[75]. While epigenetic effects on X chromosome genes could affect risk for autism, this hypothesis has not yet been empirically tested. In summary, at present it appears that there are X-linked causes of ASC, but these represent a far smaller percentage of cases than is seen in learning difficulties.

Girls with Turner Syndrome (TS) (characterized by the XO karyotype) [76] are at an increased risk for ASC, which could be the result of an X-linked recessive gene, but this is not clear-cut since XYY and XXYY males are also at increased risk [77]. One study [78] has also reported higher autistic traits scores (as measured on the Autism Spectrum Quotient [AQ] in XXY males), though this is not always seen [77].

There are other possible versions of the X chromosome theory of ASC. Although females have two X chromosomes, only one of these is generally active. *X chromosome inactivation* (the process by which one X chromosome is suppressed while the other remains active) acts to negate the “dosage” difference in X chromosome genes between males and females. However, 10%–15% of X chromosome genes may continue to be expressed from the supposedly inactive X. Gong and colleagues [79] directly tested this hypothesis and found no evidence for a skewed X chromosome inactivation in a large sample of individuals with and without ASC. X chromosome gene dosage could play a role in sex ratios if the non-silenced genes were protective. However, comparing the incidence of ASC across different sex aneuploidies does not suggest a simple dosage effect, and frequently the ASC occurs in the context of clear learning disabilities, and so could simply be secondary to the latter. It is increasingly recognized that learning difficulties are themselves a risk factor for ASC [80], so any evaluation of the X chromosome theory needs to consider these separately.

*Genomic imprinting* (the process by which genetic effects are influenced by whether the genes are transmitted through the father or the mother [81]) is also of interest. Ordinarily this would not result in sex differences in the rate of a condition, but could do so if the imprinting affects the X chromosome. Skuse [82],[83] suggested that an imprinted X-locus could explain sex differences in social and communication skills and the male vulnerability to social and communication impairment. His theory was inspired by the finding that in individuals with TS, the rate of social difficulties varied according to whether their single X chromosome was inherited from the father (X_p_O cases) or the mother (X_m_O cases) (where _p_ is paternal, and _m_ is maternal) [82]. Social problems are greater in X_m_O relative to X_p_O individuals. Typical females always inherit an X chromosome from both parents (X_p_X_m_), but typical males always have only a maternal X (X_m_Y). Skuse hypothesized that a gene expressed on the paternal X acts as a protective factor against the social problems seen in TS and, by extrapolation, as a protective factor against ASC.

Creswell and colleagues [84] subsequently reported five cases of ASC from an unselected sample of 150 subjects with TS. All the cases were X_m_O (or had a structurally abnormal paternal X). All of the cases in that report also had moderate to severe learning difficulties and low verbal IQ scores, despite the fact that intelligence is usually in the average range in TS. This raises the possibility that the kind of ASC observed was related to learning difficulties (i.e., applicable only to classic autism rather than the full autistic spectrum, which includes AS). Also, given that 77% of TS females are X_m_O, while only 23% are X_p_O [85], this means that *by chance* one would expect to find ASC more often associated with X_m_O than with X_p_O.

No specific X-linked genes have yet been identified which explain these findings, but there is evidence that whichever genes are involved may modulate amygdala circuits which are disrupted in ASC [86]. Whilst the amygdala has not been directly examined, a study of the whole brain in a mouse model of TS did not identify any paternally expressed X-linked genes, but did identify a maternally expressed gene, *xlr3b*, which was implicated in cognitive flexibility [87]. However, it is unclear if a functioning human orthologue of this gene exists.

A recent study searched for imprinted genes in the preoptic area (POA) and medial prefrontal cortex (mPFC) in mouse. No X-linked imprinted genes were identified when using a cut-off of *p*<0.05, but using a less stringent cut-off of 0.1, a small set of putative X-linked imprinted genes were identified including three paternally expressed genes in the POA and three different paternally expressed genes in the mPFC [88]. Three of these genes (*cask*, *acsl4*, and *ids*) have human orthologues whose disruption can cause MR. Another intriguing finding from this study was that total levels of expression from X_m_ were increased relative to those of X_p_ in females. This could reflect preferential inactivation of the X_p_ and would act to minimize dosage differences between the sexes. If a screen of females with ASC identified rare mutations or CNVs on the X_p_, this would provide important evidence for the theory.

### The Y Chromosome Theory

Since the XYY and XXYY syndromes have an increased incidence of ASC [89]–[91], it is important to consider if the male bias in ASC could also result from the male-limited expression of genes on the Y chromosome. This possibility has attracted very little research attention. Such genes should be located in the non-recombining region of the Y. *SRY* (the sex determining gene) is expressed in the medial rostral hypothalamus, as well as the frontal and temporal regions of the human brain [92]. In vitro assays suggest that *SRY* can increase transcription of tyrosine hydroxylase (the rate-limiting enzyme in dopamine biosynthesis) by binding at a promoter site [93]. In addition, the knockdown of *SRY* expression in the substantia nigra of the rat decreases tyrosine hydroxylase expression [94]. This could implicate SRY in the male bias for disorders involving disregulated catecholamines such as ADHD. SRY may also regulate the monoamine oxidase A (*MAO-A*) gene [95]. Other Y-linked genes known to be expressed in human brain include *ZFY* and *PCDH11Y* [92],[96].

A small candidate gene study failed to find associations between variants in *PCDH11Y* and autism [96], while *ZFY* has not been specifically investigated. One study has reported a missense variant in *NLGN4Y* in a single patient with autism and his father with learning difficulties [97]. Comparison of Y chromosome haplotype groups between cases and controls represents an alternative strategy to identifying Y chromosome effects. Two such studies have been conducted in regard to ASC—one was positive [98] and one was negative [99]. Y chromosome effects certainly merit additional research attention, but current evidence is too sparse to evaluate to what extent this mechanism could explain the sex bias in ASC.

### Reduced Autosomal Penetrance in Females? A Final Theory

For completeness we briefly mention a final theory, arising from studies of rare CNVs with ASC [67],[74],[100],[101]. As mentioned earlier, these scans have *not* routinely implicated the X chromosome, but this final model proposes that a significant proportion of ASC cases are the result of dominant de novo mutations (on the autosomes) which have reduced penetrance in females. Statistical analysis of ASC family data has provided supporting evidence [102]. A problem for this theory, however, is that the majority of studies report that the sex ratio in children with ASC and de novo CNVs is 1∶1. This clearly does not fit with reduced penetrance in females [103]. A second problem for this theory is that it does not address *why* penetrance should be reduced in females. However, we agree that it is critical that large-scale linkage and association studies test for sex-specific effects.

### Not Mutually Exclusive Theories

The X and Y chromosome theories and the fT model offer potential explanations for the biased sex ratio in ASC and warrant further research. While often conceived as competing theories, they need not be mutually exclusive. This is because we cannot rule out the possibility that genes on the X and Y chromosomes may be regulated by fT or have products that affect the production or sensitivity of an individual to fT. X chromosome genes may also regulate Y chromosome genes and vice versa. In addition, it is possible that X or Y chromosome genes and fT exposure are independent risk factors for ASC.

The theories do, however, make contrasting predictions for individuals with certain intersex conditions, in particular those with Complete Androgen Insensitivity Syndrome (CAIS), where there is a complete deficiency of working androgen receptors, in the presence of a typical male genetic complement (XY). Given the rarity of this condition, studies using measures of autistic traits (such as the AQ [104]) may be more feasible than studies of diagnosed cases of ASC in CAIS per se. (These contrasting predictions are summarized in Table 5.)

**Table 5:** Rates of ASC/autistic traits in different medical conditions, as predicted by the X and Y chromosome theories, and the fT theory.

Medical Condition	Prediction from X-Dosage or X-Linked Recessive Model	Prediction from Imprinted X Model	Prediction from Y-Chromosome Model	Prediction from FT Theory
Complete Androgen Insensitivity Syndrome (CAIS) in males	Similar to typical males	Similar to typical males	Similar to typical males	Similar to typical females
Congenital Adrenal Hyperplasia (CAH) in females	Similar to typical females	Similar to typical females	Similar to typical females	Similar to typical males
Turner Syndrome (with a maternal X; X_m_O)	Similar to typical males	Similar to typical males	Similar to typical females	Similar to typical females
Turner Syndrome (with a paternal X; X_p_O)	Similar to typical males	Similar to typical females	Similar to typical females	Similar to typical females

Finally, whilst it may be that the psychiatric classification system is “carving nature at its joints,” it is also possible that some of the underlying hormonal and genetic mechanisms are involved not just in ASC but are relevant to a broader category of neurodevelopmental conditions (see Box 1).

**Box 1.** fT and X-linked factors in other neurodevelopmental conditions.

**ADHD:** fT has been implicated by several studies using the proxy measure of 2D∶4D (finger) ratio [176],[184],[185] and one study of genetic variation at the androgen receptor [186]. An animal model of ADHD suggests that early androgen exposure affects catecholamine innervation of the frontal cortex and cognitive function [187]. ADHD has also been associated with X-linked genes, in particular monoamine oxidase-B [188],[189] and steroid sulfatase [190]. The latter has also been implicated in attention deficits in a mouse model of Turner Syndrome [191]. However, genome-wide scans have not implicated the X chromosome in ADHD [192],[193].

**Conduct Disorder (CD):** Activational effects of gonadal steroids have shown relationships with CD [194]–[196], but there is not a simple one-to-one correspondence. In addition, the X-linked gene coding for monoamine oxidase A has been linked to aggression and neural hyperactivity to threat [197].

**Reading Disorder/Dyslexia:** Two studies have failed to find a relation between 2D∶4D (digit) ratio (as a proxy for fT) and dyslexia [115],[198]. One genome-wide linkage analysis suggested a locus on Xq26 [199]. A nearby susceptibility locus in a single extended family has also been reported [198].

**Specific Language Impairment:** The correlation between amniotic fT levels and early vocabulary [46],[200] could indicate a role for fT in SLI. Genome-wide linkage studies have not implicated the X chromosome [201]–[203].

**Tourette Syndrome:** Tics in individuals with TS increase in intensity during puberty, suggesting an activational testosterone effect. A role for fT has also been proposed based on a study of gender dysphoria, play preferences, and spatial skills in individuals with TS [204]. Genome-wide linkage studies have not implicated the X chromosome [205], but Lawson-Yuen [206] have reported a pedigree with a NLGN4X deletion which was associated with TS in one family-member.

## Looking Ahead: Toward a Unified Theory?

For as long as ASC has been recognized, a higher prevalence has been observed in males, yet until 1997, when our group proposed the extreme male brain theory, this potential clue to the etiology of the condition went unexplored [105]. In the early years following the publication of the EMB theory, the majority of the evidence relevant to the theory came from psychological studies, but since 2001 supporting evidence has also come from biology.

In the present article we have considered studies that suggest that fetal testosterone is involved in sex differences in key areas of behavior and cognition in the general population (in social development, language development, empathy, systemizing, and attention to detail), as well as in influencing brain structure, and the number of autistic traits an individual possesses. Understanding the relationship between empathy and systemizing will require more research because presenting them as independent ignores the fact that both are related to fT. Nor can we yet extrapolate the fT results to individuals with an ASC diagnosis since this will require much larger collections of amniotic samples than has been possible to date. Strengthening a role for fT in ASC is the recent genetic evidence in which SNPs in key sex steroid genes are associated with either diagnosed AS and/or autistic traits.

The main alternatives to the fT theory are the X and Y chromosome theories. Future research could usefully test these theories against each other, or test if all are valid, either independently or because of gene-hormone interactions. Whilst it remains a possibility that the male bias in ASC simply reflects diagnostic difficulties in recognizing ASC in females, the link between ASC and maleness has generated a novel framework for exploring the link between sex and ASC, and a wealth of data relating prenatal hormones to masculinization of the mind and the brain.

## Supporting Information

Text S1 Supplementary material.
(DOC)

## Abbreviations

ADHD: Attention Deficit and Hyperactivity Disorder
AQ: Autism Spectrum Quotient
AS: Asperger Syndrome
ASC: Autism Spectrum Conditions
CAIS: Complete Androgen Insensitivity Syndrome
CD: conduct disorder
CNV: copy number variation
EMB: extreme male brain
EQ: Empathy Quotient
E-S: Empathizing-Systemizing
fT: fetal testosterone
mPFC: medial prefrontal cortex
nT: neonatal testosterone
POA: preoptic area
SQ: Systemizing Quotient
TS: Turner Syndrome

## References

[1] A.P.A (1994). DSM-IV diagnostic and statistical manual of mental disorders, 4th edition.

[2] Baird G, Simonoff E, Pickles A, Chandler S, Loucas T (2006). Prevalence of disorders of the autism spectrum in a population cohort of children in South Thames: the Special Needs and Autism Project (SNAP).. Lancet.

[3] Baron-Cohen S, Scott F. J, Allison C, Williams J. G, Bolton P (2009). Autism spectrum prevalence: a school-based U.K. population study.. Brit J Psychiat.

[4] Bauman M. L, Kemper T. L (2005). Neuroanatomic observations of the brain in autism: a review and future directions.. Int J Dev Neurosci.

[5] Stodgell C. J, Ingram J. I, Hyman S. L (2001). The role of candidate genes in unravelling the genetics of autism.. Int Rev Res Ment Ret.

[6] Geschwind D. H (2008). Autism: many genes, common pathways?. Cell.

[7] Fombonne E (2005). The changing epidemiology of autism.. J Appl Res Intellect.

[8] Chakrabarti S, Fombonne E (2001). Pervasive developmental disorders in pre-school children.. JAMA-J Am Med Assoc.

[9] Gillberg C, Cederlund M, Lamberg K, Zeijlon L (2006). Brief report: “the autism epidemic”. The registered prevalence of autism in a Swedish urban area.. J Autism Dev Disord.

[10] Rutter M, Caspi A, Moffitt T. E (2003). Using sex differences in psychopathology to study causal mechanisms: unifying issues and research strategies.. J Child Psychol Psyc.

[11] Treasure J. L (2007). Getting beneath the phenotype of anorexia nervosa: the search for viable endophenotypes and genotypes.. Can J Psychiat.

[12] New A. S, Triebwasser J, Charney D. S (2008). The case for shifting borderline personality disorder to Axis I.. Biol Psychiat.

[13] Holliday-Willey L (1999). Pretending to be normal.

[14] Baron-Cohen S (2003). The essential difference: men, women and the extreme male brain..

[15] Baron-Cohen S, Wheelwright S (2004). The Empathy Quotient (EQ). An investigation of adults with Asperger Syndrome or High Functioning Autism, and normal sex differences.. J Autism Dev Disord.

[16] Baron-Cohen S, Richler J, Bisarya D, Gurunathan N, Wheelwright S (2003). The Systemising Quotient (SQ): an investigation of adults with Asperger Syndrome or High Functioning Autism and normal sex differences.. Philos T Roy Soc B.

[17] Wheelwright S, Baron-Cohen S, Goldenfeld N, Delaney J, Fine D (2006). Predicting Autism Spectrum Quotient (AQ) from the Systemizing Quotient-Revised (SQ-R) and Empathy Quotient (EQ).. Brain Res.

[18] Auyeung B, Baron-Cohen S, Chapman E, Knickmeyer R, Taylor K (2006). Foetal testosterone and the Child Systemizing Quotient (SQ-C).. Eur J Endrocrinol.

[19] Baron-Cohen S, Knickmeyer R, Belmonte M (2005). Sex differences in the brain: implications for explaining autism.. Science.

[20] Gilmore J. H, Lin W, Prastawa M. W, Looney C. B, Vetsa Y. S (2007). Regional gray matter growth, sexual dimorphism, and cerebral asymmetry in the neonatal brain.. J Neurosci.

[21] Courchesne E, Campbell K, Solso S (2010). Brain growth across the life span in autism: age-specific changes in anatomical pathology.. Brain Res.

[22] Good C. D, Johnsrude I, Ashburner J, Henson R. N, Friston K. J (2001). Cerebral asymmetry and the effects of sex and handedness on brain structure: a voxel-based morphometric analysis of 465 normal adult human brains.. Neuroimage.

[23] Schumann C. M, Hamstra J, Goodlin-Jones B. L, Lotspeich L. J, Kwon H (2004). The amygdala is enlarged in children but not adolescents with autism; the hippocampus is enlarged at all ages.. J Neurosci.

[24] Schumann C. M, Barnes C. C, Lord C, Courchesne E (2009). Amygdala enlargement in toddlers with autism related to severity of social and communication impairments.. Biol Psychiat.

[25] Mosconi M. W, Cody-Hazlett H, Poe M. D, Gerig G, Gimpel-Smith R (2009). Longitudinal study of amygdala volume and joint attention in 2- to 4-year-old children with autism.. Arch Gen Psychiat.

[26] Baron-Cohen S, Ring H, Wheelwright S, Bullmore E. T, Brammer M. J (1999). Social intelligence in the normal and autistic brain: an fMRI study.. Eur J Neurosci.

[27] Ring H, Baron-Cohen S, Williams S, Wheelwright S, Bullmore E (1999). Cerebral correlates of preserved cognitive skills in autism. A functional MRI study of Embedded Figures task performance.. Brain.

[28] Baron-Cohen S, Ring H, Chitnis X, Wheelwright S, Gregory L (2006). fMRI of parents of children with Asperger Syndrome: a pilot study.. Brain Cognition.

[29] Manjaly Z. M, Bruning N, Neufang S, Stephan K. E, Brieber S (2007). Neurophysiological correlates of relatively enhanced local visual search in autistic adolescents.. Neuroimage.

[30] Geschwind N, Galaburda A. M (1985). Cerebral lateralization: biological mechanisms, associations and pathology. III. A hypothesis and a program for research.. Arch Neurol-Chicago.

[31] Phoenix C. H, Goy R. W, Gerall A. A, Young W. C (1959). Organizing action of prenatally administered testosterone propionate on the tissues mediating mating behavior in the female guinea pig.. Endocrinology.

[32] Arnold A. P, Breedlove S. M (1985). Organizational and activational effects of sex steroids on brain and behavior: a reanalysis.. Horm Behav.

[33] De Vries G, Simerley R. B, Pfaff D. W, Arnold A. P, Etgen A. M, Fahrbach S. E, Moss R. L (2002). Anatomy, development and function of sexually dimorphic neural circuits in the mammalian brain.. Hormones, brain and behaviour: development of hormone-dependent neuronal systems.

[34] Baron-Cohen S, Lutchmaya S, Knickmeyer R (2004). Prenatal testosterone in mind: amniotic fluid studies.

[35] Collaer M, Hines M (1995). Human behavioural sex differences: a role for gonadal hormones during early development?. Psychol Bull.

[36] Hines M (2004). Brain gender.

[37] Smail P. J, Reyes F. I, Winter J. S. D, Fairman C, Kogan S. J, Hafez E. S. E (1981). The fetal hormonal environment and its effect on the morphogenesis of the genital system.. Pediatric andrology.

[38] van de Beek C, Thijssen J. H. H, Cohen-Kettenis P. T, van Goozen S. H. M, Buitelaar J. K (2004). Relationships between sex hormones assessed in amniotic fluid, and maternal and umbilical cord serum: what is the best source of information to investigate the effects of fetal hormonal exposure?. Horm Behav.

[39] Money J, Ehrhardt A. A (1972). Man and woman, boy and girl.

[40] Dawood M. Y (1977). Hormones in amniotic fluid.. Am J Obstet Gynecol.

[41] Finegan J. A, Bartleman B, Wong P. Y (1989). A window for the study of prenatal sex hormone influences on postnatal development.. J Genet Psychol.

[42] Judd H. L, Robinson J. D, Young P. E, Jones O. W (1976). Amniotic fluid testosterone levels in midpregnancy.. Obstet Gynecol.

[43] Nagami M, McDonough P, Ellegood J, Mahesh V (1979). Maternal and amniotic fluid steroids throughout human pregnancy.. Am J Obstet Gynaecol.

[44] Robinson J. D, Judd H. L, Young P. E, Jones O. W, Yen S. S (1977). Amniotic fluid androgens and estrogens in midgestation.. J Clin Endocrin Metab.

[45] Lutchmaya S, Baron-Cohen S, Raggatt P (2002). Foetal testosterone and eye contact in 12 month old infants.. Infant Behav and Dev.

[46] Lutchmaya S, Baron-Cohen S, Raggatt P (2002). Foetal testosterone and vocabulary size in 18- and 24-month-old infants.. Infant Behav and Dev.

[47] Knickmeyer R, Baron-Cohen S, Raggatt P, Taylor K (2005). Foetal testosterone, social cognition, and restricted interests in children.. J Child Psychol Psych.

[48] Chapman E, Baron-Cohen S, Auyeung B, Knickmeyer R, Taylor K (2006). Foetal testosterone and empathy: evidence from the Empathy Quotient (EQ) and the ‘Reading the Mind in the Eyes’ Test.. Soc Neurosci.

[49] Knickmeyer R, Baron-Cohen S, Raggatt P, Taylor K, Hackett G (2006). Foetal testosterone and empathy.. Horm Behav.

[50] Auyeung B, Ashwin E, Knickmeyer R, Taylor K, Hackett G Effects of fetal testosterone on visiospatial ability..

[51] Chura L. R, Lombardo M. V, Ashwin E, Auyeung B, Chakrabarti B (2010). Organizational effects of fetal testosterone on human corpus callosum size and asymmetry.. Psychoneuroendocrinology.

[52] Auyeung B, Taylor K, Hackett G, Baron-Cohen S (2010). Foetal testosterone and autistic traits in 18 to 24-month-old children.. Mol Autism.

[53] Auyeung B, Baron-Cohen S, Ashwin E, Knickmeyer R, Taylor K (2009). Fetal testosterone and autistic traits.. Brit J Psychol.

[54] Nguyen D. K, Disteche C. M (2006). High expression of the mammalian X chromosome in brain.. Brain Res.

[55] Laumonnier F, Cuthbert P. C, Grant S. G (2007). The role of neuronal complexes in human X-linked brain diseases.. Am J Hum Genet.

[56] Gecz J, Shoubridge C, Corbett M (2009). The genetic landscape of intellectual disability arising from chromosome X.. Trends Genet.

[57] Ropers H. H, Hamel B. C (2005). X-linked mental retardation.. Nat Rev Genet.

[58] Bailey D. B, Raspa M, Olmsted M, Holiday D. B (2008). Co-occurring conditions associated with FMR1 gene variations: findings from a national parent survey.. Am J Med Genet Part A.

[59] Consortium IMGSoA (1998). A full genome screen for autism with evidence for linkage to a region on chromosome 7q.. Hum Mol Genet.

[60] Hallmayer J, Pintado E, Lotspeich L, Spiker D, McMahon W (1994). Molecular analysis and test of linkage between the FMR-1 gene and infantile autism in multiplex families.. Am J Hum Genet.

[61] Hallmayer J, Hebert J. M, Spiker D, Lotspeich L, McMahon W. M (1996). Autism and the X chromosome. Multipoint sib-pair analysis.. Arch Gen Psychiat.

[62] Risch N, Spiker D, Lotspeich L, Nouri N, Hinds D (1999). A genomic screen of autism: evidence for a multilocus etiology.. Am J Hum Genet.

[63] Schutz C. K, Polley D, Robinson P. D, Chalifoux M, Macciardi F (2002). Autism and the X chromosome: no linkage to microsatellite loci detected using the affected sibling pair method.. Am J Hum Genet.

[64] Schellenberg G. D, Dawson G, Sung Y. J, Estes A, Munson J (2006). Evidence for multiple loci from a genome scan of autism kindreds.. Mol Psychiat.

[65] Duvall J. A, Lu A, Cantor R. M, Todd R. D, Constantino J. N (2007). A quantitative trait locus analysis of social responsiveness in multiplex autism families.. Am J Psychiat.

[66] Kilpinen H, Ylisaukko-oja T, Rehnstrom K, Gaal E, Turunen J. A (2009). Linkage and linkage disequilibrium scan for autism loci in an extended pedigree from Finland.. Hum Mol Genet.

[67] Szatmari P, Paterson A. D, Zwaigenbaum L, Roberts W, Brian J (2007). Mapping autism risk loci using genetic linkage and chromosomal rearrangements.. Nat Genet.

[68] Philippe A, Martinez M, Guilloud-Bataille M, Gillberg C, Rastam M (1999). Genome-wide scan for autism susceptibility genes. Paris Autism Research International Sibpair Study.. Hum Mol Genet.

[69] Wang K, Zhang H, Ma D, Bucan M, Glessner J. T (2009). Common genetic variants on 5p14.1 associate with autism spectrum disorders.. Nature.

[70] Weiss L. A, Arking D. E, Daly M. J, Chakravarti A (2009). A genome-wide linkage and association scan reveals novel loci for autism.. Nature.

[71] Auranen M, Vanhala R, Varilo T, Ayers K, Kempas E (2002). A genome-wide screen for autism-spectrum disorders: evidence for a major susceptibility locus on chromosome 3q25-27.. Am J Hum Genet.

[72] Shao Y, Wolpert C. M, Raiford K. L, Menold M. M, Donnelly S. L (2002). Genomic screen and follow-up analysis for autistic disorder.. Am J Med Genet.

[73] Morrow E. M, Yoo S. Y, Flavell S. W, Kim T. K, Lin Y (2008). Identifying autism loci and genes by tracing recent shared ancestry.. Science.

[74] Sebat J, Lakshmi B, Malhotra D, Troge J, Lese-Martin C (2007). Strong association of de novo copy number mutations with autism.. Science.

[75] Weiss L. A, Shen Y, Korn J. M, Arking D. E, Miller D. T (2008). Association between microdeletion and microduplication at 16p11.2 and autism.. New Engl J Med.

[76] Lippe B (1991). Turner syndrome.. Endocrinol Metab Clin.

[77] Tartaglia N. R, Hansen R. L, Reynolds A, Hessl D, Bacalman S (2006). Attention deficit hyperactivity disorder and autism spectrum disorders in males with XXY, XYY and XXYY syndromes.. J Intell Disabil Res.

[78] van Rijn S, Swaab H, Aleman A, Kahn R. S (2008). Social behavior and autism traits in a sex chromosomal disorder: Klinefelter (47XXY) syndrome.. J Autism Dev Disord.

[79] Gong X, Bacchelli E, Blasi F, Toma C, Betancur C (2008). Analysis of X chromosome inactivation in autism spectrum disorders.. Am J Med Genet Part B: Neuropsychiatric Genetics.

[80] Wing L, Gould J (1979). Severe impairments of social interaction and associated abnormalities in children: epidemiology and classification.. J Autism Dev Disord.

[81] Keverne E. B (1997). Genomic imprinting in the brain.. Curr Opin Neurobiol.

[82] Skuse D. H, James R. S, Bishop D. V. M, Coppins B, Dalton P (1997). Evidence from Turner's syndrome of the imprinted X-linked locus affecting cognitive function.. Nature.

[83] Skuse D. H (2000). Imprinting, the X-chromosome, and the male brain: explaining sex differences in the liability to autism.. Ped Res.

[84] Creswell C. S, Skuse D. H (1999). Autism in association with Turner Syndrome: genetic implications for male vulnerability to pervasive developmental disorders.. Neurocase.

[85] Grumbach M. M, Hughes I. A, Conte F. A, Larsen P. R (2003). Williams textbook of endocrinology.. Williams textbook of endocrinology.

[86] Skuse D (2006). Genetic influences on the neural basis of social cognition.. Philos T Roy Soc B.

[87] Davies W, Isles A, Smith R, Karunadasa D, Burrmann D (2005). Xlr3b is a new imprinted candidate for X-linked parent-of-origin effects on cognitive function in mice.. Nat Genet.

[88] Gregg C, Zhang J, Butler J. E, Haig D, Dulac C (2010). Sex-specific parent-of-origin allelic expression in the mouse brain.. Science.

[89] Bruining H, Swaab H, Kas M, van Engeland H (2009). Psychiatric characteristics in a self-selected sample of boys with Klinefelter syndrome.. Pediatrics.

[90] Geerts M, Steyaert J, Fryns J. P (2003). The XYY syndrome: a follow-up study on 38 boys.. Genet Counsel.

[91] Tartaglia N, Davis S, Hench A, Nimishakavi S, Beauregard R (2008). A new look at XXYY syndrome: medical and psychological features.. Am J Med Genet Part A.

[92] Mayer A, Lahr G, Swaab D. F, Pilgrim C, Reisert I (1998). The Y-chromosomal genes SRY and ZFY are transcribed in adult human brain.. Neurogenetics.

[93] Milsted A, Serova L, Sabban E. L, Dunphy G, Turner M. E (2004). Regulation of tyrosine hydroxylase gene transcription by Sry.. Neurosci Lett.

[94] Dewing P, Chiang C. W, Sinchak K, Sim H, Fernagut P. O (2006). Direct regulation of adult brain function by the male-specific factor SRY.. Curr Biol.

[95] Wu J. B, Chen K, Li Y, Lau Y. F, Shih J. C (2009). Regulation of monoamine oxidase A by the SRY gene on the Y chromosome.. FASEB J.

[96] Durand C. M, Kappeler C, Betancur C, Delorme R, Quach H (2006). Expression and genetic variability of PCDH11Y, a gene specific to Homo sapiens and candidate for susceptibility to psychiatric disorders.. Am J Med Genet Part B: Neuropsychiatric Genetics.

[97] Yan J, Feng J, Schroer R, Li W, Skinner C (2008). Analysis of the neuroligin 4Y gene in patients with autism.. Psychiatr Genet.

[98] Serajee F. J, Mahbubul Huq A. H (2009). Association of Y chromosome haplotypes with autism.. J Child Neurol.

[99] Jamain S, Quach H, Betancur C, Rastam M, Colineaux C (2003). Mutations of the X-linked genes encoding neuroligins NLGN3 and NLGN4 are associated with autism.. Nat Genet.

[100] Christian S. L, Brune C. W, Sudi J, Kumar R. A, Liu S (2008). Novel submicroscopic chromosomal abnormalities detected in autism spectrum disorder.. Biol Psychiat.

[101] Marshall C. R, Noor A, Vincent J. B, Lionel A. C, Feuk L (2008). Structural variation of chromosomes in autism spectrum disorder.. Am J Hum Genet.

[102] Zhao X, Leotta A, Kustanovich V, Lajonchere C, Geschwind D. H (2007). A unified genetic theory for sporadic and inherited autism.. P Natl Acad Sci U S A.

[103] Beaudet A. L (2007). Autism: highly heritable but not inherited.. Nat Med.

[104] Baron-Cohen S, Wheelwright S, Skinner R, Martin J, Clubley E (2001). The Autism Spectrum Quotient (AQ) evidence from Asperger Syndrome/High Functioning Autism, males and females, scientists and mathematicians.. J Autism Dev Disord.

[105] Baron-Cohen S (2002). The extreme male brain theory of autism.. Trends Cogn Sci.

[106] Arnold L (1999). Sex differences in ADHD: conference summary.. J Abnorm Child Psych.

[107] Biederman J, Mick E, Faraone S. V, Braaten E, Doyle A (2002). Influence of gender on attention deficit hyperactivity disorder in children referred to a psychiatric clinic.. Am J Psychiat.

[108] Bauermeister J. J, Shrout P. E, Chavez L, Rubio-Stipec M, Ramirez R (2007). ADHD and gender: are risks and sequela of ADHD the same for boys and girls?. J Child Psychol Psyc.

[109] Costello E. J, Mustillo S, Erkanli A, Keeler G, Angold A (2003). Prevalence and development of psychiatric disorders in childhood and adolescence.. Arch Gen Psychiat.

[110] Merikangas K. J, He J, Brody D, Fisher P, Bourdon K (2010). Prevalence and treatment of mental disorders among US children in the 2001–2004 NHANES.. Pediatrics.

[111] Simon V, Czobor P, Balint S, Meszaros A, Bitter I (2009). Prevalence and correlates of adult attention-deficit hyperactivity disorder: meta-analysis.. Brit J Psychiat.

[112] Loeber R, Burke J. D, Lahey B. B, Winters A, Zera M (2000). Oppositional defiant and conduct disorder: a review of the past 10 years, part I.. J Am Acad Child Psy.

[113] Moffitt T. E, Caspi A (2001). Childhood predictors differentiate life-course persistent and adolescence-limited antisocial pathways among males and females.. Dev and Psychopathol.

[114] Shaywitz S. E, Shaywitz B. A, Fletcher J. M, Escobar M. D (1990). Prevalence of reading disability in boys and girls. Results of the Connecticut Longitudinal Study.. JAMA-J Am Med Assoc.

[115] Liederman J, Kantrowitz L, Flannery K (2005). Male vulnerability to reading disability is not likely to be a myth: a call for new data.. J Learn Disabil.

[116] Bishop D. V. M (1997). Uncommon understanding: development and disorders of language comprehension in children.

[117] Tomblin J. B, Records N. L, Buckwalter P, Zhang X, Smith E (1997). Prevalence of specific language impairment in kindergarten children.. J Speech Lang Hear R.

[118] Law J, Rush R, Schoon I, Parsons S (2009). Modeling developmental language difficulties from school entry into adulthood: literacy, mental health, and employment outcomes.. J Speech Lang Hear R.

[119] Kadesjo B, Gillberg C (2000). Tourette's disorder: epidemiology and comorbidity in primary school children.. J Am Acad Child Psy.

[120] Baron-Cohen S, Hoekstra R. A, Knickmeyer R, Wheelwright S (2006). The Autism-Spectrum Quotient (AQ)-Adolescent version.. J Autism Dev Disord.

[121] Wakabayashi A, Baron-Cohen S, Wheelwright S (2004). The Autism Spectrum Quotient (AQ) Japanese version: evidence from high-functioning clinical group and normal adults.. Japan J Psychol.

[122] Wakabayashi A, Baron-Cohen S, Wheelwright S, Tojo Y (2006). The Autism-Spectrum Quotient (AQ) in Japan: a cross-cultural comparison.. J Autism Dev Disord.

[123] Wakabayashi A, Baron-Cohen S, Uchiyama T, Yoshida Y, Tojo Y (2007). The Autism-Spectrum Quotient (AQ) Children's Version in Japan: a cross-cultural comparison.. J Autism Dev Disord.

[124] Hoekstra R, Bartels M, Cath D. C, Boomsma D. I (2008). Factor structure, reliability and criterion validity of the Autism-Spectrum Quotient (AQ): a study in Dutch population and patient groups.. J Autism Dev Disord.

[125] Auyeung B, Baron-Cohen S, Wheelwright S, Allison C (2008). The Autism Spectrum Quotient: Children's Version (AQ-Child).. J Autism Dev Disord.

[126] Auyeung B, Wheelwright S, Allison C, Atkinson M, Samarawickrema N (2009). The children's Empathy Quotient and Systemizing Quotient: sex differences in typical development and in autism spectrum conditions.. J Autism Dev Disord.

[127] Scott F, Baron-Cohen S, Bolton P, Brayne C (2002). Prevalence of autism spectrum conditions in children aged 5–11 years in Cambridgeshire, UK.. Autism.

[128] Scott F, Baron-Cohen S, Bolton P, Brayne C (2002). The CAST (Childhood Asperger Syndrome Test) preliminary development of UK screen for mainstream primary-school children.. Autism.

[129] Williams J, Allison C, Scott F, Bolton P, Baron-Cohen S (2008). The Childhood Autism Spectrum Test (CAST): sex differences.. J Autism Dev Disord.

[130] Williams J, Scott F. J, Allison C, Bolton P, Baron-Cohen S (2005). The CAST (Childhood Asperger Syndrome Test): test accuracy.. Autism.

[131] Shah A, Frith U (1983). An islet of ability in autism: a research note.. J Child Psychol Psyc.

[132] Jolliffe T, Baron-Cohen S (1997). Are people with autism or Asperger's Syndrome faster than normal on the Embedded Figures Task?. J Child Psychol Psyc.

[133] Lawson J, Baron-Cohen S, Wheelwright S (2004). Empathising and systemising in adults with and without Asperger Syndrome.. J Autism Dev Disord.

[134] Baron-Cohen S, Wheelwright S, Scahill V, Lawson J, Spong A (2001). Are intuitive physics and intuitive psychology independent?. J Dev Learn Dis.

[135] Constantino J. N, Todd R. D (2003). Autistic traits in the general population.. Arch Gen Psychiat.

[136] Constantino J. N, Todd R. D (2005). Intergenerational transmission of subthreshold autistic traits in the general population.. Biol Psychiat.

[137] Allison C, Baron-Cohen S, Wheelwright S, Charman T, Richler J (2008). The Q-CHAT (Quantitative Checklist for Autism in Toddlers): a normally distributed quantitative measure of autistic traits at 18–24 months of age: preliminary report.. J Autism Dev Disord.

[138] Baron-Cohen S, O'Riordan M, Jones R, Stone V, Plaisted K (1999). A new test of social sensitivity: detection of faux pas in normal children and children with Asperger syndrome.. J Autism Dev Disord.

[139] Baron-Cohen S, Wheelwright S (2003). The Friendship Questionnaire (FQ): an investigation of adults with Asperger Syndrome or High Functioning Autism, and normal sex differences.. J Autism Dev Disord.

[140] Baron-Cohen S, Jolliffe T, Mortimore C, Robertson M (1997). Another advanced test of theory of mind: evidence from very high functioning adults with autism or Asperger Syndrome.. J Child Psychol Psyc.

[141] Redcay E, Courchesne E (2005). When is the brain enlarged in autism? A meta-analysis of all brain size reports.. Biol Psychiat.

[142] Hazlett H. C, Poe M, Gerig G, Smith R. G, Provenzale J (2005). Magnetic resonance imaging and head circumference study of brain size in autism: birth through age 2 years.. Arch Gen Psychiat.

[143] Courchesne E, Carper R, Akshoomoff N. A (2003). Evidence of brain overgrowth in the first year of life in autism.. JAMA-J Am Med Assoc.

[144] Cheng Y, Chou K. H, Decety J, Chen I. Y, Hung D (2009). Sex differences in the neuroanatomy of human mirror-neuron system: a voxel-based morphometric investigation.. Neurosci.

[145] Yamasue H, Abe O, Suga M, Yamada H, Rogers M. A (2008). Sex-linked neuroanatomical basis of human altruistic cooperativeness.. Cereb Cortex.

[146] Giedd J. N, Viatuzis A. C, Hamburger S. D, Lange N, Rajapakse J. C (1996). Quantitative MRI of the temporal lobe, amygdala and hippocampus in normal human development: ages 4–18 years.. J Comp Neurol.

[147] Goldstein J. M, Seidman L. J, Horton N. J, Makris N, Kennedy D. N (2001). Normal sexual dimorphism of the adult human brain assessed by *in vivo* Magnetic Resonance Imaging.. Cereb Cortex.

[148] Wilke M, Krageloh-Mann I, Holland S. K (2007). Global and local development of gray and white matter volume in normal children and adolescents.. Exp Brain Res.

[149] Peper J. S, Brouwer R. M, Schnack H. G, van Baal G. C, van Leeuwen M (2008). Cerebral white matter in early puberty is associated with luteinizing hormone concentrations.. Psychoneuroendocrinol.

[150] Chen X, Sachdev P. S, Wen W, Anstey K. J (2007). Sex differences in regional gray matter in healthy individuals aged 44–48 years: a voxel-based morphometric study.. Neuroimage.

[151] Lenroot R. K, Gogtay N, Greenstein D. K, Wells E. M, Wallace G. L (2007). Sexual dimorphism of brain developmental trajectories during childhood and adolescence.. Neuroimage.

[152] Frazier T. W, Hardan A. Y (2009). A meta-analysis of the corpus callosum in autism.. Biol Psychiat.

[153] Rojas D. C, Bawn S. D, Benkers T. L, Reite M. L, Rogers S. J (2002). Smaller left hemisphere planum temporale in adults with autistic disorder.. Neurosci Lett.

[154] Rojas D. C, Camou S. L, Reite M. L, Rogers S. J (2005). Planum temporale volume in children and adolescents with autism.. J Autism Dev Disord.

[155] Witelson S. F, Glezer I. I, Kigar D. L (1995). Women have greater density of neurons in posterior temporal cortex.. J Neurosci.

[156] Sowell E. R, Peterson B. S, Kan E, Woods R. P, Yoshii J (2007). Sex differences in cortical thickness mapped in 176 healthy individuals between 7 and 87 years of age.. Cereb Cortex.

[157] Witelson S. F, Kigar D. L (1992). Sylvian fissure morphology and asymmetry in men and women: bilateral differences in relation to handedness in men.. J Comp Neurol.

[158] Herbert M. R, Ziegler D. A, Deutsch C. K, O'Brien L. M, Kennedy D. N (2005). Brain asymmetries in autism and developmental language disorder: a nested whole-brain analysis.. Brain.

[159] Wada J. A, Clarke R, Hamm A (1975). Cerebral hemispheric asymmetry in humans. Cortical speech zones in 100 adults and 100 infant brains.. Arch Neurol-Chicago.

[160] Gage N. M, Juranek J, Filipek P. A, Osann K, Flodman P (2009). Rightward hemispheric asymmetries in auditory language cortex in children with autistic disorder: an MRI investigation.. J Neurodev Disord.

[161] Im K, Lee J. M, Lee J, Shin Y. W, Kim I. Y (2006). Gender difference analysis of cortical thickness in healthy young adults with surface-based methods.. Neuroimage.

[162] Luders E, Narr K. L, Thompson P. M, Rex D. E, Woods R. P (2006). Gender effects on cortical thickness and the influence of scaling.. Hum Brain Mapp.

[163] Brun C. C, Lepore N, Luders E, Chou Y. Y, Madsen S. K (2009). Sex differences in brain structure in auditory and cingulate regions.. Neuroreport.

[164] Hadjikhani N, Joseph R. M, Snyder J, Tager-Flusberg H (2006). Anatomical differences in the mirror neuron system and social cognition network in autism.. Cereb Cortex.

[165] McAlonan G. M, Cheung V, Suckling J, Lam G. Y, Tai K. S (2005). Mapping the brain in autism: a voxel based MRI study of volumetric differences and intercorrelations in autism.. Brain.

[166] Biswal B. B, Mennes M, Zuo X. N, Gohel S, Kelly C (2010). Toward discovery science of human brain function.. P Natl Acad Sci U S A.

[167] Kennedy D. P, Courchesne E (2008). The intrinsic functional organization of the brain is altered in autism.. Neuroimage.

[168] Lee P. S, Foss-Feig J, Henderson J. G, Kenworthy L. E, Gilotty L (2007). Atypical neural substrates of Embedded Figures Task performance in children with Autism Spectrum Disorder.. Neuroimage.

[169] Chakrabarti B, Dudbridge F, Kent L, Wheelwright S, Hill-Cawthorne G (2009). Genes related to sex steroids, neural growth, and social-emotional behavior are associated with autistic traits, empathy, and Asperger syndrome.. Autism Res.

[170] Tordjman A, Ferrari P, Sulmont V, Duyme M, Roubertoux P (1997). Androgenic activity in autism.. Am J Psychiat.

[171] Knickmeyer R, Baron-Cohen S (2006). Foetal testosterone and sex differences in typical social development and in autism.. J Child Neurol.

[172] Ingudomnukul E, Baron-Cohen S, Knickmeyer R, Wheelwright S (2007). Elevated rates of testosterone-related disorders in a sample of women with autism spectrum conditions.. Horm Behav.

[173] Knickmeyer R, Wheelwright S, Baron-Cohen S. B (2008). Sex-typical play: masculinization/defeminization in girls with an autism spectrum condition.. J Autism Dev Disord.

[174] Manning J, Baron-Cohen S, Wheelwright S, Sanders G (2001). Autism and the ratio between 2nd and 4th digit length.. Dev Med Child Neurol.

[175] Milne E, White S, Campbell R, Swettenham J, Hansen P (2006). Motion and form coherence detection in Autistic Spectrum Disorder: relationship to motor control and 2∶4 digit ratio.. J Autism Dev Disord.

[176] de Bruin E. I, Verheij F, Wiegman T, Ferdinand R. F (2006). Differences in finger length ratio between males with autism, pervasive developmental disorder-not otherwise specified, ADHD, and anxiety disorders.. Dev Med Child Neurol.

[177] Henningsson S, Jonsson L, Ljunggren E, Westberg L, Gillberg C (2009). Possible association between the androgen receptor gene and autism spectrum disorder.. Psychoneuroendocrinol.

[178] Hu V. W, Nguyen A, Kim K. S, Steinberg M. E, Sarachana T (2009). Gene expression profiling of lymphoblasts from autistic and nonaffected sib pairs: altered pathways in neuronal development and steroid biosynthesis.. PLoS ONE.

[179] Sarachana T, Xu M, Wu R. C, Hu V. W (2011). Sex hormones in autism: androgens and estrogens differentially and reciprocally regulate RORA, a novel candidate gene for autism.. PLoS ONE.

[180] Nguyen A, Rauch T. A, Pfeifer G. P, Hu V. W (2010). Global methylation profiling of lymphoblastoid cell lines reveals epigenetic contributions to autism spectrum disorders and a novel autism candidate gene, RORA, whose protein product is reduced in autistic brain.. FASEB J.

[181] Knickmeyer R, Baron-Cohen S, Fane B. A, Wheelwright S, Mathews G. A (2006). Androgens and autistic traits: a study of individuals with congenital adrenal hyperplasia.. Horm Behav.

[182] Schmidtova E, Kelemenova S, Celec P, Ficek A, Ostatnikova D (2010). Polymorphisms in genes involved in testosterone metabolism in Slovak autistic boys.. Endocrinologist.

[183] Ruta L, Ingudomnukul E, Taylor K, Chakrabarti B, Baron-Cohen S (2011). Increased serum androstenedione in adults with autism spectrum conditions.. Psychoneuroendocrinol.

[184] McFadden D, Westhafer J. G, Pasanen E. G, Carlson C. L, Tucker D. M (2005). Physiological evidence of hypermasculinization in boys with the inattentive subtype of attention-deficit/hyperactivity disorder (ADHD).. Clin Neurosci Res.

[185] Martel M. M, Gobrogge K. L, Breedlove S. M, Nigg J. T (2008). Masculinized finger-length ratios of boys, but not girls, are associated with attention-deficit/hyperactivity disorder.. Behav Neurosci.

[186] Comings D. E, Chen C, Wu S, Muhleman D (1999). Association of the androgen receptor gene (AR) with ADHD and conduct disorder.. Neuroreport.

[187] King J. A, Barkley R. A, Delville Y, Ferris C. F (2000). Early androgen treatment decreases cognitive function and catecholamine innervation in an animal model of ADHD.. Behav Brain Res.

[188] Jiang S, Xin R, Wu X, Lin S, Qian Y (2000). Association between attention deficit hyperactivity disorder and the DXS7 locus.. Am J Medical Genet.

[189] Rommelse N. N, Altink M. E, Arias-Vasquez A, Buschgens C. J, Fliers E (2008). Differential association between MAOA, ADHD and neuropsychological functioning in boys and girls.. Am J Medical Genet Part B Neuropsychiatric Genetics.

[190] Brookes K. J, Hawi Z, Kirley A, Barry E, Gill M (2008). Association of the steroid sulfatase (STS) gene with attention deficit hyperactivity disorder.. Am J Medical Genet Part B Neuropsychiatric Genetics.

[191] Davies W, Humby T, Isles A. R, Burgoyne P. S, Wilkinson L. S (2007). X-monosomy effects on visuospatial attention in mice: a candidate gene and implications for Turner syndrome and attention deficit hyperactivity disorder.. Biol Psychiat.

[192] Fisher S. E, Francks C, McCracken J. T, McGough J. J, Marlow A. J (2002). A genomewide scan for loci involved in attention-deficit/hyperactivity disorder.. Am J Hum Genet.

[193] Franke B, Neale B. M, Faraone S. V (2009). Genome-wide association studies in ADHD.. Hum Genet.

[194] Pajer K, Tabbah R, Gardner W, Rubin R. T, Czambel R. K (2006). Adrenal androgen and gonadal hormone levels in adolescent girls with conduct disorder.. Psychoneuroendocrinol.

[195] Rowe R, Maughan B, Worthman C. M, Costello E. J, Angold A (2004). Testosterone, antisocial behaviour and social dominance in boys: pubertal development and biosocial interaction.. Biol Psychiat.

[196] Dorn L. D, Kolko D. J, Susman E. J, Huang B, Stein H (2009). Salivary gonadal and adrenal hormone differences in boys and girls with and without disruptive behavior disorders: contextual variants.. Biol Psychiat.

[197] Meyer-Lindenberg A, Buckholtz J. W, Kolachana B, R. H. A, Pezawas L (2006). Neural mechanisms of genetic risk for impulsivity and violence in humans.. P Natl Acad Sci U S A.

[198] van Gelder M, Tijms J, Hoeks J (2005). Second to fourth digit ratio and dyslexia: no evidence for an association between reading disabilities and the 2D∶4D ratio.. Dev Med Child Neurol.

[199] Fisher S. E, Francks C, Marlow A. J, MacPhie I. L, Newbury D. F (2002). Independent genome-wide scans identify a chromosome 18 quantitative-trait locus influencing dyslexia.. Nat Genet.

[200] Finegan J. K, Niccols G. A, Sitarenios G (1992). Relations between prenatal testosterone levels and cognitive abilities at 4 years.. Dev Psychol.

[201] Bartlett C. W, Flax J. F, Logue M. W, Vieland V. J, Bassett A. S (2002). A major susceptibility locus for specific language impairment is located on 13q21.. Am J Hum Genet.

[202] Villanueva P, Newbury D. F, Jara L, De Barbieri Z, Mirza G (2011). Genome-wide analysis of genetic susceptibility to language impairment in an isolated Chilean population.. Eur J HumGenet Jan.

[203] Newbury D. F, Ishikawa-Brush Y, Marlow A. J, Fisher S. E, Monaco A. P (2002). A genomewide scan identifies two novel loci involved in specific language impairment.. Am J Hum Genet.

[204] Alexander G. M, Peterson B. S (2004). Testing the prenatal hormone hypothesis of tic-related disorders: gender identity and gender role behavior.. Dev Psychopathol.

[205] State M. W (2010). The genetics of child psychiatric disorders: focus on autism and Tourette syndrome.. Neuron.

[206] Lawson-Yuen A, Saldivar J. S, Sommer S, Picker J (2008). Familial deletion within NLGN4 associated with autism and Tourette syndrome.. Eur J Hum Genet.

