# Supplementary material for: Why Are Autism Spectrum Conditions More Prevalent in Males?
Source: PLoS Biol. 2011 Jun 14;9(6):e1001081. doi: 10.1371/journal.pbio.1001081 (PMC3114757; doi:10.1371/journal.pbio.1001081)
Supplement: Text S1 — Supplementary material. (DOC) [file pbio.1001081.s001.doc]

**Supplementary Material** to *Why Are Autism Spectrum Conditions*

*More Prevalent in Males?*

Simon Baron-Cohen*, Michael V. Lombardo*, Bonnie Auyeung*, Emma Ashwin*, Bhismadev Chakrabarti*+, and Rebecca Knickmeyer*++.

**Sex Differences in Empathy and Systemizing and the EMB theory**

Individuals with ASC score lower than typical males on the ‘Reading the Mind in the Eyes’ task [1], the Social Stories Questionnaire [2], the Friendship and Relationship Questionnaire (which tests the importance of emotional intimacy and sharing in relationships) [3] and on tests of recognizing complex emotions from videos of facial expressions or audios of vocalizations [4]. Individuals with ASC have intact or superior functioning on tests of intuitive physics [2,5], a domain which shows a sex difference in favor of males [2]. Individuals with ASC are faster and more accurate than controls on the Embedded Figures Task (EFT), a task on which typical males perform better than typical females [6,7]. The EFT requires good attention to detail, a prerequisite for systemizing.

Additional evidence for the EMB theory comes from measures of autistic traits. On the Childhood Autism Spectrum Test (CAST) [8,9] boys score higher than girls [10], and children with ASC score higher than controls [11]. On the Autism Spectrum Quotient (AQ) [12] individuals with ASC score higher than those without a diagnosis [12] and the same has been found on the child and adolescent versions of this instrument [13,14], as well as on a toddler measure of autistic traits [15]. Among controls, males score higher than females [12,13,14] a finding that has been reported cross-culturally [16,17,18,19]. Similar results have been found using the Social Responsiveness Scale (SRS) [20] on which individuals with an ASC diagnosis score higher than typical males, who in turn score higher than typical females [21].

**Sex Differences in the Brain and the EMB theory**

*Longitudinal Studies*

Longitudinal MRI studies demonstrate maturational changes in human brain development across the lifespan that show clear sex differences. Males have larger brains than females, a difference already apparent approximately 2 weeks after birth and that persists even when controlling for differences in birth weight [22]. Infant males possess approximately 10% more gray matter (GM), 6% more white matter (WM), and 7% larger subcortical volumes than infant females [22]. Early in life, infants and toddlers with autism show even more pronounced brain size that typical males. Evidence for this comes from studies showing increased head circumference [23], and GM and WM enlargement throughout cortex [24], within the first years of life in ASC. A recent longitudinal MRI study in infants and toddlers shows that the typical male enlargement of frontal and temporal GM is more pronounced in ASC [25]. Between the ages of 4-20 years old, typical males continue to possess more white matter (WM), and WM grows in a linear fashion within both sexes [26]. During adolescence, WM growth has a steeper trajectory in males than females [27]. Gray matter (GM) in contrast, matures in a nonlinear (cubic or quadratic) fashion across most of cortex between the ages of 4-33 years old [28]. The age at which GM reaches its peak size differs in a lobe-specific manner across frontal, parietal, and temporal cortex, with males peaking later than females [26,27]. A similar pattern of protracted neural development in males is observed in the cerebellum [29].

Because males generally have larger brains than do females, this gross difference in brain size is a potential confound in studies assessing neural sexual dimorphism. For this reason it is important to either consider differences between the sexes after controlling for total brain volume, or in samples where males and females are matched on total brain volume. When looking at the data in this way, some striking regionally-specific sex differences appear: Frontal GM (but not WM) and corpus callosum volume are proportionally increased in size in females relative to males, after accounting for total brain volume. In contrast, occipital GM and WM are proportionally increased in size in males relative to females [27]. Within the cerebellum, the superior and inferior lobes are increased in size in males than females, while there are no differences in the size of the anterior lobe or corpus medullare [29].

So far, the lack of longitudinal studies in autism in the age ranges of 4 years through to adulthood limit what is known about whether such sexual dimorphism is exaggerated in autism. Thus, when longitudinal studies across the lifespan in autism are conducted, we will be able to assess whether exaggerations of sexual dimorphism in these particular ways persist past early infancy and childhood.

*Cross-Sectional Studies*

While longitudinal investigations are important in characterizing neural sexual dimorphism across the lifespan, these studies have been limited to gross aggregate measurements across large regions of cortex. This limits the scope for pinpointing exactly where regionally specific proportional differences in size between the sexes may exist. However, several cross-sectional voxel-based morphometry (VBM) and cortical thickness studies of neural sexual dimorphism have been reported which help pinpoint exactly where such sexual dimorphism is found.

In the largest VBM study to date, assessing 465 adults ages 17-79 years old, males have proportionally larger amygdalae, cerebellum (near the superior lobule), and left temporal pole. In contrast, females have proportionally larger orbitofrontal and cingulate cortex as well as lateral fronto-parietal-temporal regions such as perisylvian language areas (Heschl’s gyrus/planum temporale), inferior frontal gyrus, and inferior parietal lobule [30]. Many replications of the amygdala and cerebellum findings in both VBM and region of interest (ROI) studies have been reported [29,31,32,33,34,35,36,37].

Similarly for females, studies consistently find increased thickness or size of cortex in females, particularly in lateral fronto-parietal cortices [31,32,34,37,38,39,40,41]. This may be because the female brain has increased cortical gyrification, particularly in lateral fronto-parietal cortices, which implies increased cortical surface area despite smaller total brain size [42].

The human brain is also markedly asymmetrical [43] and some anatomical asymmetries are more pronounced in males. The most robust sexual dimorphism in anatomical asymmetry is in perisylvian language areas such as Heschl’s gyrus and planum temporale. This area is typically larger in the left hemisphere than the right [44] and this Left>Right asymmetry is larger in males than in females [30,45,46].

Of these more localized examples of sexual dimorphism in brain structure, which are exaggerated in autism? The amygdala is substantially enlarged early in development (independent of total brain volume) in ASC [47,48,49]. The corpus callosum (CC) is consistently identified as smaller in individuals with ASC [50], and this is consistent with the finding that the CC is proportionally larger in typical females [27]. Perisylvian language areas such as Heschl’s gyrus/planum temporale are typically proportionally larger in females and are smallest in ASC [51,52]. Similarly, the pattern of Left>Right asymmetry within planum temporale is highly exaggerated within ASC [53]. Finally, females tend to have a larger lateral fronto-parietal cortex [38,40,42] and in ASC there is evidence of proportional lateral fronto-parietal cortical thinning [54] or reduction in GM density [55] in later development (post-childhood). Few studies have compared controls to ASC using samples of both males and females, but those that do consistently report more pronounced atypical neurodevelopment of these regions in females with ASC [25,48,56,57]. However, these studies suffer from small sample sizes in females. This points to the need for future research to test sex differences in controls and ASC within the same study using larger samples.

**Sex Differences in Brain Function and the EMB theory**

The ‘default mode network’ (DMN) is decreased in functional connectivity in males relative to females during resting conditions [58]. In ASC, connectivity within the DMN is even more decreased during rest [59,60,61,62] (see Figure 1). Using task-related fMRI, typical males show decreased activity in the posterior parietal cortex (BA 7) during the Embedded Figures Test (EFT) [63] and people with ASC show even less activity in BA 7 during this task [64,65,66] (see Figure 2).

Finally, typical males show decreased activity bilaterally in the inferior frontal gyrus (BA 44/45) during the ‘Reading the Mind in the Eyes’ Test relative to typical females [63], and people with ASC show even less activity in this region during this task [67] (see Figure 3). Comparing across Figures 1-3 we see the predicted pattern [Females > Males > Autism]. In addition, mothers and fathers of children with ASC also show hyper-masculinization of brain activity during the EFT and Eyes tasks [63], suggesting that this neuroimaging phenotype is partially genetic. Whether this is true of all parents of children with ASC, or just the sub-group with the ‘Broader Autism Phenotype’ [68] needs to be clarified.

**Figure 1:** *Exaggerated sexual dimorphism using fMRI in the Default Mode Network (DMN).*

*(A) Females show stronger connectivity within the DMN compared to males [58].*

*(B) Reduced DMN connectivity in ASC [59].*


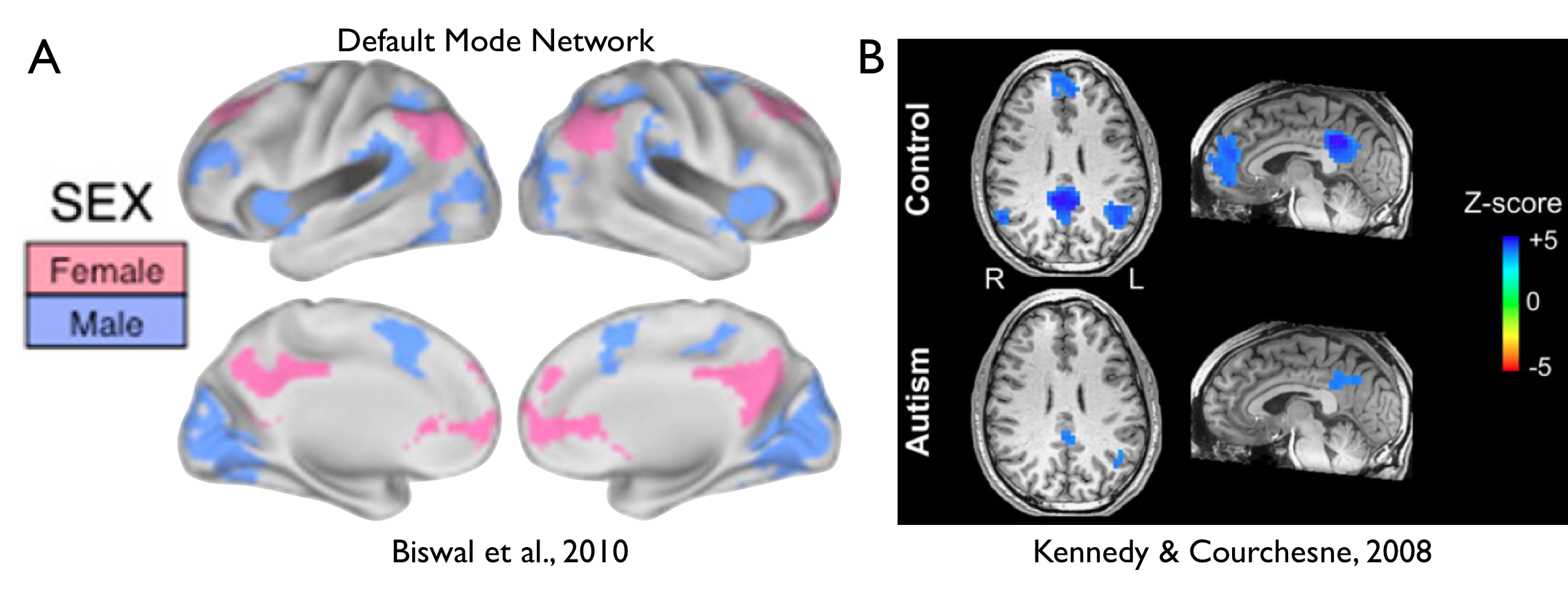


**Figure 2:** *Exaggerated sexual dimorphism using fMRI on the Embedded Figures Test (EFT).*

*(A) An example of the EFT.*

*(B) Females show greater activity than males during the EFT in posterior parietal cortex [63].*

*(C) Reduced activation in posterior parietal cortex in ASC during the EFT [64,65,66].*

*
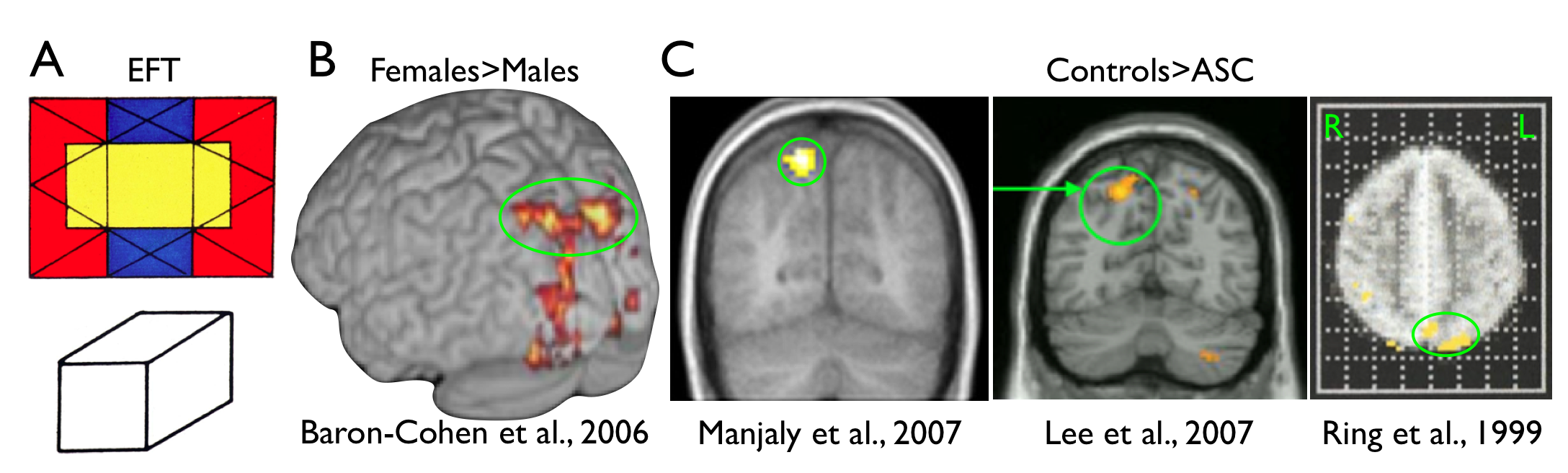
*

**Figure 3:** *Exaggerated sexual dimorphism using fMRI on the ‘Reading the Mind in Eyes’ (Eyes) test.*

*(A) An example of the Eyes task.*

*(B) Females show greater activity than males in inferior frontal gyrus during the Eyes task [63].*

*(C) Reduced activation in inferior frontal gyrus in ASC during the Eyes task [67]*.


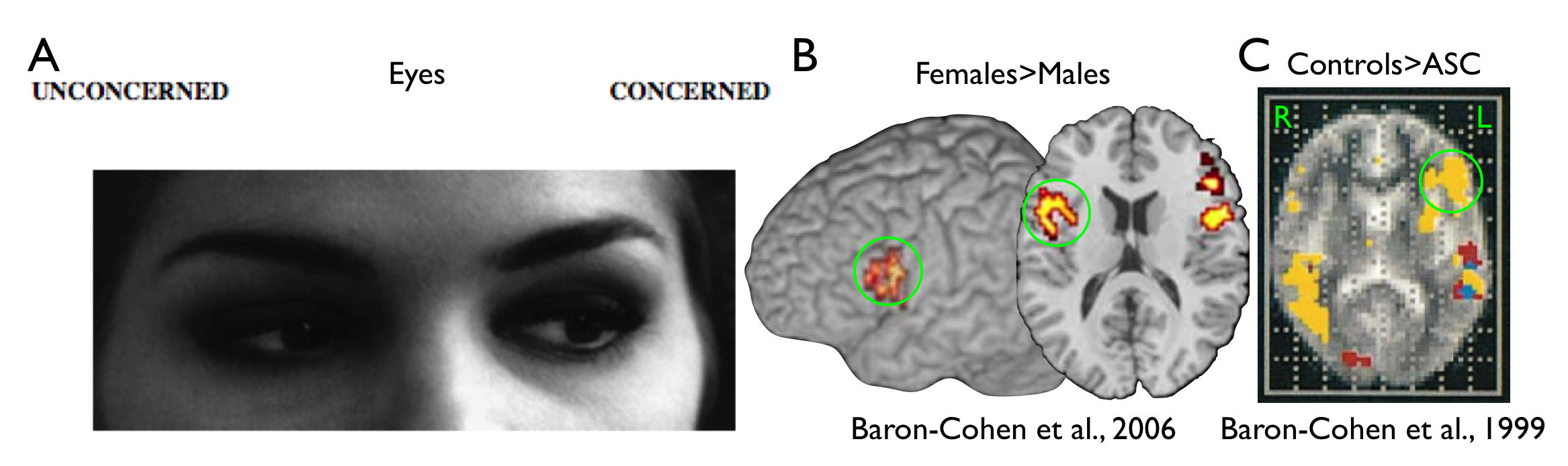


**The effects of fetal testosterone on the animal brain**

Animal experiments enable the manipulation of testosterone levels through castration (since testosterone is produced in males by the testes) or through injecting testosterone into the pregnant dam or the neonate. The neonatal testosterone surge in rats (the most commonly used model organism) is generally considered to be developmentally equivalent to the 2^nd^ trimester surge in humans since rats are born at an earlier stage of development compared to humans.

Such studies have taught us much about the effects of early testosterone exposure. For example, fetal testosterone (fT) modulates apoptosis in the sexually dimorphic nucleus of the preoptic area (SDN-POA) within the hypothalamus [69,70], the anteroventral periventricular nucleus (AVPV) located in the periventricular gray area at the rostral extreme of the third ventricle [71,72]. Neonatal testosterone (nT) (manipulated at birth) also modulates apoptosis in the sexually dimorphic nucleus of the preoptic area [69] and in a sexually dimorphic nucleus of motoneurons called the spinal nucleus of the bulbocavernosus [73] [74,75]. nT also promotes the differentiation of vasopressin-expressing cells in the sexually dimorphic bed nucleus of the stria terminalis (BNST) and medial amygdaloid nucleus [76], and modulates dendritic spine density and astrocytic complexity in the arcuate nucleus [77]. Androgen and estrogen receptors are densely expressed throughout these regions as well as important subcortical structures implicated in autism, such as the amygdala, and hippocampus [78] Manipulation of fT and nT levels influences many aspects of the neuroanatomical sexual dimorphism in the amygdala and hippocampus [76,79,80,81,82,83,84].

Sex steroid receptors also exist in many areas of the cerebral cortex [78]. However, given the much smaller cerebral cortices in species such as rats and mice, where most of the studies have focused on, relatively few studies have explored fT and/or nT effects on the cerebral cortex. Furthermore, despite several studies on the behavioral effects of fT exposure on rhesus macaques [85,86], no reports on their neuroanatomy have yet been published. This is an important area for future research as rhesus macaques and humans share some sexually dimorphic features in brain development, such as overall larger brain volume in males, proportionately larger WM volumes in males, and proportionately greater volumes of the putamen, caudate, and hippocampus in females [87]. Thus, studies on nonhuman primates and their correspondence to studies of fT-effects on sexual dimorphisms in the cerebral cortex of humans will be an exciting new avenue for extending knowledge on how early exposure to androgens affects later expression of sexual dimorphism in the brain.

**Fetal androgens affect ASC traits: evidence from rare medical conditions**

The most widely used medical model of fT effects on behavior is *Congenital Adrenal Hyperplasia (CAH)*, a condition in which an enzymatic defect (usually caused by mutations in the gene coding for 21-hydroxylase (*CYP21*), or the 11-beta hydroxylase (*CYP11B1*), results in exposure to high levels of adrenal androgens, beginning very early in gestation. Prenatal androgen exposure is in the normal male range [88]. It has an estimated incidence of 1 in 15,000 live births [89].

The model has been validated by the consistent finding that females with CAH are more interested in male-typical activities and are less interested in female-typical activities, relative to unaffected females [90,91,92,93,94]. Females with CAH have been reported to score higher than sex-matched controls on spatial orienting, mental rotation and targeting, which could reflect enhanced systemizing [95,96,97,98]. The magnitude of these effects is in the range of 0.5 to 1 standard deviations (r values from 0.23 to 0.36). It has also been suggested that females with CAH have lower empathy, intimacy, and desire for close social relationships, though this may reflect other factors [99,100,101,102]. The magnitude of these effects varies widely with the test used so more targeted tests are clearly needed.

Of more relevance to the EMB and fT theories of ASC, girls with CAH also have a higher number of autistic traits on the Autism Spectrum Quotient (AQ) relative to their unaffected sisters [103] (a difference of 0.5 SD, r = 0.24). It should also be noted that CAH is usually diagnosed shortly after birth and treatment instituted which returns androgens to a normal female level. Thus, if the nT surge is implicated in risk for ASC, CAH will not be a fully appropriate model.

A second example of a rare medical condition affecting response to fT is *Complete Androgen Insensitivity Syndrome (CAIS)*. It occurs when there is a complete deficiency of working androgen receptors. It is an X-linked recessive disorder and hence occurs more often in chromosomal males. Prevalence is approximately 1 in 20,000 live male births [89]. At birth, chromosomally male infants with CAIS are phenotypically female, despite their XY complement, and are therefore usually raised as girls, given girls’ names and develop a female gender identity. At puberty, breasts develop under the influence of estrogen derived from testicular androgens. The testes remain undescended, their existence unknown to the individual and their families, but producing testosterone. In the absence of any functioning androgen receptors the individual appears indistinguishable from typical females in outward appearance. Diagnosis usually takes place in adolescence when menarche fails to occur. The discovery of the Y chromosome in such individuals comes as a huge shock psychologically, and the majority of such individuals choose to continue living with their female identity, not disclosing their AIS [89,104]. No study has yet examined ASC traits in this group (predicted to be lower than in typical XY males if autistic traits are influenced by fT) or ASC diagnosis (predicted to be lower than typical XY males), in part because CAIS is rare. Such a study would be highly complementary to existing work in females with CAH.

**Androgens and ASC: proxy measures of fT, and current hormones**

Regarding proxy measures of fT, children with ASC have lower second digit to fourth digit (2D:4D) ratios than typically developing children (the difference is between 1 and 2 SD, r ≈ 0.53) [105,106,107]. 2D:4D ratio is lower in men than in women. Sex differences in 2D:4D ratio are apparent by week 14 of fetal life [108] and 2D:4D ratio is influenced by fT [109]. This suggests that children with ASC have been exposed to higher levels of prenatal androgens.

Regarding current hormones, androgen-related medical conditions (such as polycystic ovary syndrome (PCOS), ovarian growths, and hirsutism) occur at elevated rates in women with ASC, and in mothers of children with ASC [110]. A subset of male adolescents with ASC also show hyper-androgeny, or elevated levels of androgens, and precocious puberty [111]. Related to this, delayed menarche has also been found in females with ASC [110,112]. While there are many potential causes for this observation, puberty timing partially reflects hormonal programming of the hypothalamic-pituitary-gonadal axis during gestation [113].

In addition, left-handedness, non-right-handedness and ambidexterity are more common in typical males [114] and even more common in individuals with ASC [115,116]. Body asymmetries are related to prenatal sex hormones, and breast or testis size on the left vs. right sides of the body are related to cognition [117], though the Geschwind and Gallaburda model that first proposed this [118] has been criticized for being over-extended [116]. fT is implicated in left-handedness and asymmetric lateralization [116,119,120,121].

**The role of sex steroid genes in ASC and/or autistic traits**

Testosterone is one of the products of a chain of biochemical reactions that synthesize several sex steroids. In the first candidate gene association study of Asperger Syndrome (AS), eight genes in this pathway were nominally associated with a diagnosis of AS. In a parallel candidate gene association study of autistic traits in the general population, five genes from this sex steroid pathway were found to be nominally associated with number of autistic traits (scores on the Autism Spectrum Quotient (AQ) and/or scores on the Empathy Quotient (EQ) [122].

Specifically, single nucleotide polymorphisms (SNPs) in the genes encoding Cytochrome P450 containing enzymes (*CYP19A1*, *CYP17A1* and *CYP11B1*) were associated with differences in allele frequency in a sample of people with AS, compared to a control group selected for low AQ. The other class of genes involved in steroidigenesis is those that code for the Hydroxysteroid dehydrogenases, which do not contain Cytochrome P450. Polymorphisms in three of these genes (*HSD11B1, HSD17B4, HSD17B2*) were nominally associated with autistic traits and/or a diagnosis of AS [122]. *CYP11B1* was additionally associated with scores on the EQ in the general population. *CYP19A1* codes for aromatase, the enzyme that catalyses the conversion of testosterone to estradiol. This may offer a crucial clue in the mechanism of action of sex steroids in the brain, since evidence from mouse models shows that testosterone is converted to estrogen in the fetal brain (through aromatase), and exerts its effects through estrogen receptors. Consistent with this, SNPs in the Estrogen Receptors (*ESR1* and *ESR2*) were associated with higher AQ scores in the general population, as well as with a diagnosis of AS. Estrogen receptor expression, both in the neonatal as well as the adult human brain, is tightly linked to expression of oxytocin and vasopressin receptors [123,124], whose role in social behavior is well established [125,126]. Another study measuring gene expression levels in siblings discordant for autism [127] reported an over expression of two genes involved in the synthesis of androgens (SCARB1 and SRD5A1) in lymphocyte cell lines derived from the siblings with a diagnosis. Finally, a recent and novel finding from the same group, implicates a novel gene, retinoic acid-related orphan receptor-alpha (RORA), to the transcription of the protein aromatase, that converts testosterone into estradiol [128]. In post-mortem frontal cortex tissue, these investigators found that expression of RORA and aromatase were highly correlated (r^2^ = 0.915). Both the expression of RORA and aromatase was reduced in post-mortem frontal cortex tissue of individuals with autism, compared to controls. In another study, RORA expression was reduced in post-mortem frontal and cerebellum tissue of individuals with autism [129]

**References**

1. Baron-Cohen S, Jolliffe T, Mortimore C, Robertson M (1997) Another advanced test of theory of mind: evidence from very high functioning adults with autism or Asperger Syndrome. Journal of Child Psychology and Psychiatry 38: 813-822.

2. Lawson J, Baron-Cohen S, Wheelwright S (2004) Empathising and systemising in adults with and without Asperger Syndrome. Journal of Autism and Developmental Disorders 34: 301-310.

3. Baron-Cohen S, Wheelwright S (2003) The Friendship Questionnaire (FQ): An investigation of adults with Asperger Syndrome or High Functioning Autism, and normal sex differences. Journal of Autism and Developmental Disorders 33: 509-517.

4. Golan O, Baron-Cohen S, Hill J (2006) The Cambridge Mindreading (CAM) Face-Voice Battery: Testing complex emotion recognition in adults with and without Asperger syndrome. Journal of Autism and Developmental Disorders 36: 169-183.

5. Baron-Cohen S, Wheelwright S, Scahill V, Lawson J, Spong A (2001) Are intuitive physics and intuitive psychology independent? Journal of Developmental and Learning Disorders 5: 47-78.

6. Shah A, Frith U (1983) An islet of ability in autism: a research note. Journal of Child Psychology and Psychiatry 24: 613-620.

7. Jolliffe T, Baron-Cohen S (1997) Are people with autism or Asperger's Syndrome faster than normal on the Embedded Figures Task? Journal of Child Psychology &Psychiatry 38: 527-534.

8. Scott F, Baron-Cohen S, Bolton P, Brayne C (2002) Prevalence of autism spectrum conditions in children aged 5-11 years in Cambridgeshire, UK. Autism 6: 231-237.

9. Scott F, Baron-Cohen S, Bolton P, Brayne C (2002) The CAST (Childhood Asperger Syndrome Test) : Preliminary development of UK screen for mainstream primary-school children. Autism 6: 9-31.

10. Williams J, Allison C, Scott F, Bolton P, Baron-Cohen S, et al. (2008) The Childhood Autism Spectrum Test (CAST): Sex Differences. Journal of Autism and Developmental Disorders 38.

11. Williams J, Scott FJ, Allison C, Bolton P, Baron-Cohen S, et al. (2005) The CAST (Childhood Asperger Syndrome Test): test accuracy. Autism 9: 45-68.

12. Baron-Cohen S, Wheelwright S, Skinner R, Martin J, Clubley E (2001) The Autism Spectrum Quotient (AQ) : Evidence from Asperger Syndrome/High Functioning Autism, Males and Females, Scientists and Mathematicians. Journal of Autism and Developmental Disorders 31: 5-17.

13. Baron-Cohen S, Hoekstra RA, Knickmeyer R, Wheelwright S (2006) The Autism-Spectrum Quotient (AQ)-Adolescent version. Journal of Autism and Developmental Disorders 36: 343-350.

14. Auyeung B, Baron-Cohen S, Wheelwright S, Allison C (2008) The Autism Spectrum Quotient: Children's Version (AQ-Child). Journal of Autism and Developmental Disorders 38: 1230-1240.

15. Allison C, Baron-Cohen S, Wheelwright S, Charman T, Richler J, et al. (2008) The Q-CHAT (Quantitative CHecklist for Autism in Toddlers): a normally distributed quantitative measure of autistic traits at 18-24 months of age: preliminary report. Journal of Autism and Developmental Disorders 38: 1414-1425.

16. Wakabayashi A, Baron-Cohen S, Wheelwright S (2004) The Autism Spectrum Quotient (AQ) Japanese version: Evidence from high-functioning clinical group and normal adults. Japanese Journal of Psychology 75: 78-84.

17. Wakabayashi A, Baron-Cohen S, Wheelwright S, Tojo Y (2006) The Autism-Spectrum Quotient (AQ) in Japan: A cross-cultural comparison. Journal of Autism and Developmental Disorders 36: 263-270.

18. Wakabayashi A, Baron-Cohen S, Uchiyama T, Yoshida Y, Tojo Y, et al. (2007) The Autism-Spectrum Quotient (AQ) Children's Version in Japan: A Cross-Cultural Comparison. Journal of Autism and Developmental Disorders 37: 491-500.

19. Hoekstra R, Bartels M, Cath DC, Boomsma D, I, (2008) Factor structure, reliability and criterion validity of the Autism-Spectrum Quotient (AQ): a study in Dutch population and patient groups. Journal of Autism and Developmental Disorders 38: 1555-1566

20. Constantino JN, Todd RD (2003) Autistic traits in the general population. Archives of General Psychiatry 60: 524-530.

21. Constantino JN, Todd RD (2005) Intergenerational transmission of subthreshold autistic traits in the general population. Biological Psychiatry 57: 655-660.

22. Gilmore JH, Lin W, Prastawa MW, Looney CB, Vetsa YS, et al. (2007) Regional gray matter growth, sexual dimorphism, and cerebral asymmetry in the neonatal brain. Journal of Neuroscience 27: 1255-1260.

23. Courchesne E, Carper R, Akshoomoff NA (2003) Evidence of Brain Overgrowth in the First Year of Life in Autism. Journal of American Medical Association 290: 337-344.

24. Hazlett HC, Poe M, Gerig G, Smith RG, Provenzale J, et al. (2005) Magnetic resonance imaging and head circumference study of brain size in autism: birth through age 2 years. Archives of General Psychiatry 62: 1366-1376.

25. Schumann CM, Bloss CS, Barnes CC, Wideman GM, Carper RA, et al. (2010) Longitudinal magnetic resonance imaging study of cortical development through early childhood in autism. Journal of Neuroscience 30: 4419-4427.

26. Giedd JN, Blumenthal J, Jeffries NO, Castellanos FX, Liu H, et al. (1999) Brain development during childhood and adolescence: a longitudinal MRI study. Nature Neuroscience 2: 861-863.

27. Lenroot RK, Gogtay N, Greenstein DK, Wells EM, Wallace GL, et al. (2007) Sexual dimorphism of brain developmental trajectories during childhood and adolescence. Neuroimage 36: 1065-1073.

28. Shaw P, Kabani NJ, Lerch JP, Eckstrand K, Lenroot R, et al. (2008) Neurodevelopmental trajectories of the human cerebral cortex. Journal of Neuroscience 28: 3586-3594.

29. Tiemeier H, Lenroot RK, Greenstein DK, Tran L, Pierson R, et al. (2010) Cerebellum development during childhood and adolescence: a longitudinal morphometric MRI study. Neuroimage 49: 63-70.

30. Good CD, Johnsrude I, Ashburner J, Henson RN, Friston KJ, et al. (2001) Cerebral asymmetry and the effects of sex and handedness on brain structure: a voxel-based morphometric analysis of 465 normal adult human brains. Neuroimage 14: 685-700.

31. Cheng Y, Chou KH, Decety J, Chen IY, Hung D, et al. (2009) Sex differences in the neuroanatomy of human mirror-neuron system: a voxel-based morphometric investigation. Neuroscience 158: 713-720.

32. Yamasue H, Abe O, Suga M, Yamada H, Rogers MA, et al. (2008) Sex-linked neuroanatomical basis of human altruistic cooperativeness. Cerebal Cortex 18: 2331-2340.

33. Giedd JN, Viatuzis AC, Hamburger SD, Lange N, Rajapakse JC, et al. (1996) Quantatitive MRI of the temporal lobe, amygdala and hippocampus in normal human development: ages 4-18 years. Journal of Comparative Neurology 366: 223-230.

34. Goldstein JM, Seidman LJ, Horton NJ, Makris N, Kennedy DN, et al. (2001) Normal sexual dimorphism of the adult human brain assessed by *In Vivo* Magnetic Resonance Imaging. Cerebral Cortex 11: 490-497.

35. Wilke M, Krageloh-Mann I, Holland SK (2007) Global and local development of gray and white matter volume in normal children and adolescents. Experimental Brain Research 178: 296-307.

36. Peper JS, Brouwer RM, Schnack HG, van Baal GC, van Leeuwen M, et al. (2008) Cerebral white matter in early puberty is associated with luteinizing hormone concentrations. Psychoneuroendocrinology 33: 909-915.

37. Chen X, Sachdev PS, Wen W, Anstey KJ (2007) Sex differences in regional gray matter in healthy individuals aged 44-48 years: a voxel-based morphometric study. Neuroimage 36: 691-699.

38. Sowell ER, Peterson BS, Kan E, Woods RP, Yoshii J, et al. (2007) Sex differences in cortical thickness mapped in 176 healthy individuals between 7 and 87 years of age. Cerebral Cortex 17: 1550-1560.

39. Im K, Lee JM, Lee J, Shin YW, Kim IY, et al. (2006) Gender difference analysis of cortical thickness in healthy young adults with surface-based methods. Neuroimage 31: 31-38.

40. Luders E, Narr KL, Thompson PM, Rex DE, Woods RP, et al. (2006) Gender effects on cortical thickness and the influence of scaling. Human Brain Mapping 27: 314-324.

41. Brun CC, Lepore N, Luders E, Chou YY, Madsen SK, et al. (2009) Sex differences in brain structure in auditory and cingulate regions. Neuroreport 20: 930-935.

42. Luders E, Narr KL, Thompson PM, Rex DE, Jancke L, et al. (2004) Gender differences in cortical complexity. Nature Neuroscience 7: 799-800.

43. Toga AW, Thompson PM (2003) Mapping brain asymmetry. Nature Reviews Neuroscience 4: 37-48.

44. Geschwind N, Levitsky W (1968) Human brain: left-right asymmetries in temporal speech region. Science 161: 186-187.

45. Wada JA, Clarke R, Hamm A (1975) Cerebral hemispheric asymmetry in humans. Cortical speech zones in 100 adults and 100 infant brains. Archives of Neurology 32: 239-246.

46. Witelson SF, Kigar DL (1992) Sylvian fissure morphology and asymmetry in men and women: bilateral differences in relation to handedness in men. Journal of Comparative Neurology 323: 326-340.

47. Schumann CM, Hamstra J, Goodlin-Jones BL, Lotspeich LJ, Kwon H, et al. (2004) The amygdala is enlarged in children but not adolescents with autism; the hippocampus is enlarged at all ages. Journal of Neuroscience 24: 6392-6401.

48. Schumann CM, Barnes CC, Lord C, Courchesne E (2009) Amygdala enlargement in toddlers with autism related to severity of social and communication impairments. Biological Psychiatry 66: 942-949.

49. Mosconi MW, Cody-Hazlett H, Poe MD, Gerig G, Gimpel-Smith R, et al. (2009) Longitudinal study of amygdala volume and joint attention in 2- to 4-year-old children with autism. Archives of General Psychiatry 66: 509-516.

50. Frazier TW, Hardan AY (2009) A meta-analysis of the corpus callosum in autism. Biological Psychiatry 66: 935-941.

51. Rojas DC, Bawn SD, Benkers TL, Reite ML, Rogers SJ (2002) Smaller left hemisphere planum temporale in adults with autistic disorder. Neuroscience Letters 328: 237-240.

52. Rojas DC, Camou SL, Reite ML, Rogers SJ (2005) Planum temporale volume in children and adolescents with autism. Journal of Autism and Developmental Disorders 35: 479-486.

53. Herbert MR, Ziegler DA, Deutsch CK, O'Brien LM, Kennedy DN, et al. (2005) Brain asymmetries in autism and developmental language disorder: a nested whole-brain analysis. Brain 128: 213-226.

54. Hadjikhani N, Joseph RM, Snyder J, Tager-Flusberg H (2006) Anatomical differences in the mirror neuron system and social cognition network in autism. Cerebal Cortex 16: 1276-1282.

55. McAlonan GM, Cheung V, Suckling J, Lam GY, Tai KS, et al. (2005) Mapping the brain in autism: a voxel based MRI study of volumetric differences and intercorrelations in autism. Brain 128: 268-276.

56. Bloss CS, Courchesne E (2007) MRI neuroanatomy in young girls with autism: a preliminary study. Journal of American Academy of Child and Adolescent Psychiatry 46: 515-523.

57. Craig MC, Zaman SH, Daly EM, Cutter WJ, Robertson DM, et al. (2007) Women with autistic-spectrum disorder: magnetic resonance imaging study of brain anatomy. British Journal of Psychiatry 191: 224-228.

58. Biswal BB, Mennes M, Zuo XN, Gohel S, Kelly C, et al. (2010) Toward discovery science of human brain function. Proceedings of the National Academy of Sciences of the United States of America 107: 4734-4739.

59. Kennedy DP, Courchesne E (2008) The intrinsic functional organization of the brain is altered in autism. Neuroimage 39: 1877-1885.

60. Assaf M, Jagannathan K, Calhoun VD, Miller L, Stevens MC, et al. (2010) Abnormal functional connectivity of default mode sub-networks in autism spectrum disorder patients. Neuroimage 53: 247-256.

61. Monk CS, Peltier SJ, Wiggins JL, Weng SJ, Carrasco M, et al. (2009) Abnormalities of intrinsic functional connectivity in autism spectrum disorders. Neuroimage 47: 764-772.

62. Weng SJ, Wiggins JL, Peltier SJ, Carrasco M, Risi S, et al. (2010) Alterations of resting state functional connectivity in the default network in adolescents with autism spectrum disorders. Brain Research 1313: 202-214.

63. Baron-Cohen S, Ring H, Chitnis X, Wheelwright S, Gregory L, et al. (2006) fMRI of parents of children with Asperger Syndrome: a pilot study. Brain Cognition 61: 122-130.

64. Ring H, Baron-Cohen S, Williams S, Wheelwright S, Bullmore E, et al. (1999) Cerebral correlates of preserved cognitive skills in autism. A functional MRI study of Embedded Figures task performance. Brain 122: 1305-1315.

65. Manjaly ZM, Bruning N, Neufang S, Stephan KE, Brieber S, et al. (2007) Neurophysiological correlates of relatively enhanced local visual search in autistic adolescents. Neuroimage 35: 283-291.

66. Lee PS, Foss-Feig J, Henderson JG, Kenworthy LE, Gilotty L, et al. (2007) Atypical neural substrates of Embedded Figures Task performance in children with Autism Spectrum Disorder. Neuroimage 38: 184-193.

67. Baron-Cohen S, Ring H, Wheelwright S, Bullmore ET, Brammer MJ, et al. (1999) Social intelligence in the normal and autistic brain: an fMRI study. European Journal of Neuroscience 11: 1891-1898.

68. Piven J, Palmer P, Jacobi D, Childress D, Arndt S (1997) Broader autism phenotype: evidence from a family history study of multiple-incidence autism familiies. American Journal of Psychiatry 154: 185-190.

69. Dodson RE, Gorski RA (1993) Testosterone propionate administration prevents the loss of neurons within the central part of the medial preoptic nucleus Journal of Neurobiology 24: 80-88.

70. Dohler KD, Coquelin A, Davis F, Hines M, Shryne JE, et al. (1984) Pre- and postnatal influence of testosterone propionate and diethylstilbestrol on differentiation of the sexually dimorphic nucleus of the preoptic area in male and female rats. Brain Research 302: 291-295.

71. Sumida H, Nishizuka M, Kano Y, Arai Y (1993) Sex differences in the anteroventral periventricular nucleus of the preoptic area and in the related effects of androgen in prenatal rats. Neuroscience Letters 151: 41-44.

72. Arai Y, Murakami S, Nishizuka M (1994) Androgen enhances neuronal degeneration in the developing preoptic area: apoptosis in the anteroventral periventricular nucleus (AVPvN-POA). Hormones and Behavior 28: 313-319.

73. Cihak R, Gutmann E, Hanzlikova V (1970) Involution and hormone-induced persistence of the M. sphincter (levator) ani in female rats. Journal of Anatomy 106: 93-110.

74. Nordeen EJ, Nordeen KW, Sengelaub DR, Arnold AP (1985) Androgens prevent normally occurring cell death in a sexually dimorphic spinal nucleus. Science 229: 671-673.

75. Freeman LM, Watson NV, Breedlove SM (1996) Androgen spares androgen-insensitive motoneurons from apoptosis in the spinal nucleus of the bulbocavernosus in rats. Hormones and Behavior 30: 424-433.

76. Wang Z, Bullock NA, De Vries GJ (1993) Sexual differentiation of vasopressin projections of the bed nucleus of the stria terminals and medial amygdaloid nucleus in rats. Endocrinology 132: 2299-2306.

77. Mong JA, Glaser E, McCarthy MM (1999) Gonadal steroids promote glial differentiation and alter neuronal morphology in the developing hypothalamus in a regionally specific manner. Journal of Neuroscience 19: 1464-1472.

78. Simerly RB, Chang C, Muramatsu M, Swanson LW (1990) Distribution of androgen and estrogen receptor mRNA-containing cells in the rat brain: an in situ hybridization study. Journal of Comparative Neurology 294: 76-95.

79. Morris JA, Jordan CL, Breedlove SM (2008) Sexual dimorphism in neuronal number of the posterodorsal medial amygdala is independent of circulating androgens and regional volume in adult rats. Journal of Comparative Neurology 506: 851-859.

80. Akhmadeev AV, Kalimullina LB (2005) Dendroarchitectonics of neurons in the posterior cortical nucleus of the amygdaloid body of the rat brain as influenced by gender and neonatal androgenization. Neuroscience and Behavioral Physiology 35: 393-397.

81. Milner TA, Loy R (1982) Hormonal regulation of axonal sprouting in the hippocampus. Brain Research 243: 180-185.

82. MacLusky NJ, Hajszan T, Prange-Kiel J, Leranth C (2006) Androgen modulation of hippocampal synaptic plasticity. Neuroscience 138: 957-965.

83. Hajszan T, MacLusky NJ, Leranth C (2008) Role of androgens and the androgen receptor in remodeling of spine synapses in limbic brain areas. Hormones and Behavior 53: 638-646.

84. Isgor C, Sengelaub DR (1998) Prenatal gonadal steroids affect adult spatial behavior, CA1 and CA3 pyramidal cell morphology in rats. Hormones and Behavior 34: 183-198.

85. Wallen K (2005) Hormonal influences on sexually differentiated behavior in nonhuman primates. Frontiers of Neuroendocrinology 26: 7-26.

86. Goy RW, Bercovitch FB, McBrair MC (1988) Behavioral masculinization is independent of genital masculinization in prenatally androgenized female rhesus macaques. Hormones and Behavior 22: 552-571.

87. Knickmeyer R, Styner M, Short SJ, Lubach GR, Kang C, et al. (2009) Maturational Trajectories of Cortical Brain Development through the Pubertal Transition: Unique Species and Sex Differences in the Monkey Revealed through Structural Magnetic Resonance Imaging. Cerebal Cortex 20: 1053-1063.

88. Carson DJ, Okuno A, Lee PA, Stetten G, Didolkar SM, et al. (1982) Amniotic fluid steroid levels. Fetuses with adrenal hyperplasia, 46,XXY fetuses, and normal fetuses. American Journal of Diseases of Children 136: 218-222.

89. Grumbach MM, Hughes IA, Conte FA (2003) Disorders of sex differentiation. In: Larsen PR, Kronenburg HM, Melmed S, Polansky KS, editors. Williams Textbook of Endocrinology. Philadelphia: Saunders.

90. Berenbaum S, Hines M (1992) Early androgens are related to childhood sex-typed toy preferences. Psychological Medicine 3: 203-206.

91. Berenbaum SA, Snyder E (1995) Early hormonal influences on childhood sex-typed activity and playmate preferences: Implications for the development of sexual orientation. Developmental Psychology 31: 31-42.

92. Berenbaum SA (1999) Effects of early androgens on sex-typed activities and interests in adolescents with congenital adrenal hyperplasia. Hormones and Behavior 35: 102-110.

93. Ehrhardt AA, Baker SW (1974) Fetal androgens, human central nervous system differentiation, and behavior sex differences. In: Freidman RC, Richart RR, Van de Wiele RL, editors. Sex differences in behavior. New York: Wiley.

94. Hines M, Brook C, Conway GS (2004) Androgen and psychosexual development: core gender identity, sexual orientation and recalled childhood gender role behavior in women and men with congenital adrenal hyperplasia (CAH). Journal of Sex Research 41: 75-81.

95. Hampson E, Rovet JF, Altmann D (1998) Spatial reasoning in children with congenital adrenal hyperplasia due to 21-hydroxylase deficiency. Developmental Neuropsychology 14: 299-320.

96. Hines M, Fane BA, Pasterski VL, Mathews GA, Conway GS, et al. (2003) Spatial abilities following prenatal androgen abnormality: targeting and mental rotations performance in individuals with congenital adrenal hyperplasia. Psychoneuroendocrinology 28: 1010-1026.

97. Resnick S, Berenbaum S, Gottesman I, Bouchard T (1986) Early hormonal influences on cognitive functioning in congenital adrenal hyperplasia. Developmental Psychology 22: 191-198.

98. Malouf MA, Migeon CJ, Carson KA, Petrucci L, Wisniewski AB (2006) Cognitive outcome in adult women affected by congenital adrenal hyperplasia due to 21-hydroxylase deficiency. Hormone Research 65: 142-150.

99. Mathews GA, Fane BA, Conway GS, Brook CG, Hines M (2009) Personality and congenital adrenal hyperplasia: possible effects of prenatal androgen exposure. Hormones and Behavior 55: 285-291.

100. Helleday J, Edman G, Ritzen EM, Siwers B (1993) Personality characteristics and platelet MAO activity in women with congenital adrenal hyperplasia (CAH). Psychoneuroendocrinology 18: 343-354.

101. Resnick SM (1982) Psychological functioning in individuals with congenital adrenal hyperplasia: early hormonal influences on cognition and personality. Minneapolis: University of Minnesota.

102. Kuhnle U, Bullinger M (1997) Outcome of congenital adrenal hyperplasia. Pediatric Surgery International 12: 511-515.

103. Knickmeyer R, Baron-Cohen S, Fane BA, Wheelwright S, Mathews GA, et al. (2006) Androgens and autistic traits: A study of individuals with congenital adrenal hyperplasia. Hormones and Behavior 50: 148-153.

104. Nordenstrom A, Servin A, Bohlin G, Larsson A, Wedell A (2002) Sex-typed toy play behavior correlates with the degree of prenatal androgen exposure assessed by CYP21 genotype in girls with congenital adrenal hyperplasia. Journal of Clinical Endocrinology & Metabolism 87: 5119-5124.

105. Manning J, Baron-Cohen S, Wheelwright S, Sanders G (2001) Autism and the ratio between 2nd and 4th digit length. Developmental Medicine and Child Neurology 43: 160-164.

106. Milne E, White S, Campbell R, Swettenham J, Hansen P, et al. (2006) Motion and Form Coherence Detection in Autistic Spectrum Disorder: Relationship to Motor Control and 2:4 Digit Ratio. Journal of Autism and Developmental Disorders 36: 1-13.

107. Falter CM, Plaisted KC, Davis G (2008) Visuo-spatial processing in autism--testing the predictions of extreme male brain theory. Journal of Autism and Developmental Disorders 38: 507-515.

108. Garn SM, Burdi AR, Babler WJ, Stinson S (1975) Early prenatal attainment of adult metacarpal-phalangeal rankings and proportions. American Journal of Physical Anthropology 43: 327-332.

109. Lutchmaya S, Baron-Cohen S, Raggatt P, Manning JT (2004) Maternal 2nd to 4th digit ratios and foetal testosterone. Early Human Development 77: 23-28.

110. Ingudomnukul E, Baron-Cohen S, Knickmeyer R, Wheelwright S (2007) Elevated rates of testosterone-related disorders in a sample of women with autism spectrum conditions. Hormones and Behavior 51: 597-604.

111. Tordjman A, Ferrari P, Sulmont V, Duyme M, Roubertoux P (1997) Androgenic Activity in Autism. American Journal of Psychiatry 154: 1626-1627.

112. Knickmeyer R, Baron-Cohen S, Hoekstra RA, Wheelwright S (2006) Age of menarche in females with autism spectrum conditions. Developmental Medicine and Child Neurology 48: 1007-1008.

113. Grumbach MM, Shaw EB (1998) Further studies on the treatment of congenital adrenal hyperplasia with cortisone: IV. Effect of cortisone and compound B in infants with disturbed electrolyte metabolism, by John F. Crigler Jr, MD, Samuel H. Silverman, MD, and Lawson Wilkins, MD, Pediatrics, 1952;10:397-413. Pediatrics 102: 215-221.

114. Peters M (1991) Sex, handedness, mathematical ability, and biological causation. Canadian Journal of Psychology 45: 415-419.

115. Gillberg C (1983) Autistic children's hand preferences: results from an epidemiological study of infantile autism. Psychiatry Research 10: 21-30.

116. McManus IC, Murray B, Doyle K, Baron-Cohen S (1992) Handedness in childhood autism shows a dissociation of skill and preference. Cortex 28: 373-381.

117. Kimura D (1999) Sex and Cognition. Cambridge, MA: MIT Press.

118. Geschwind N, Galaburda AM (1985) Cerebral lateralization: biological mechanisms, associations and pathology. III. A hypothesis and a program for research. Archives of Neurology 42: 634-654.

119. Fein D, Waterhouse L, Lucci D, Pennington B, Humes M (1985) Handedness and cognitive functions in pervasive developmental disorders. Journal of Autism and Developmental Disorders 15: 323-333.

120. Satz P, Soper H, Orsini D, Henry R, Zvi J (1985) Handedness subtypes in autism. Psychiatric Annals 15: 447-451.

121. Soper H, Satz P, Orsini D, Henry R, Zvi J, et al. (1986) Handedness patterns in autism suggests subytpes. Journal of Autism and Developmental Disorders 16: 155-167.

122. Chakrabarti B, Dudbridge F, Kent L, Wheelwright S, Hill-Cawthorne G, et al. (2009) Genes related to sex steroids, neural growth, and social-emotional behavior are associated with autistic traits, empathy, and Asperger syndrome. Autism Research 2: 157-177.

123. Han TM, De Vries GJ (2003) Organizational effects of testosterone, estradiol, and dihydrotestosterone on vasopressin mRNA expression in the bed nucleus of the stria terminalis. Journal of Neurobiology 54: 502-510.

124. Perry AN, Paramadilok A, Cushing BS (2009) Neonatal oxytocin alters subsequent estrogen receptor alpha protein expression and estrogen sensitivity in the female rat. Behavioural Brain Research 205: 154-161.

125. Francis DD, Champagne FC, Meaney MJ (2000) Variations in maternal behaviour are associated with differences in oxytocin receptor levels in the rat. Journal of Neuroendocrinology 12: 1145-1148.

126. Lim MM, Young LJ (2004) Vasopressin-dependent neural circuits underlying pair bond formation in the monogamous prairie vole. Neuroscience 125: 35-45.

127. Hu VW, Nguyen A, Kim KS, Steinberg ME, Sarachana T, et al. (2009) Gene expression profiling of lymphoblasts from autistic and nonaffected sib pairs: altered pathways in neuronal development and steroid biosynthesis. PLoS One 4: e5775.

128. Sarachana T, Xu M, Wu RC, Hu VW (2011) Sex Hormones in Autism: Androgens and Estrogens Differentially and Reciprocally Regulate RORA, a Novel Candidate Gene for Autism. PLoS One 6: e17116.

129. Nguyen A, Rauch TA, Pfeifer GP, Hu VW (2010) Global methylation profiling of lymphoblastoid cell lines reveals epigenetic contributions to autism spectrum disorders and a novel autism candidate gene, RORA, whose protein product is reduced in autistic brain. The FASEB Journal 24: 3036-3051.
